# Nutritional-environmental trade-offs in potato storage and processing for a sustainable healthy diet

**DOI:** 10.1038/s41538-023-00237-8

**Published:** 2023-12-07

**Authors:** Aubin Payne, Ebenezer M. Kwofie, Prince Agyemang, Jamie I. Baum

**Affiliations:** 1 Department of Biological and Agricultural Engineering, University of Arkansas, 203 Engineering Hall, Fayetteville, AR 72701 USA; 2 Bioresource Engineering Department, McGill University, Ste-Anne-de-Bellevue, H9X 3V9 Quebec, Canada; 3 Department of Food Science, University of Arkansas, 2650 N. Young Ave, Fayetteville, AR 72704 USA; 4 Center for Human Nutrition, University of Arkansas System Division of Agriculture, Fayetteville, AR 72704 USA

**Keywords:** Sustainability, Environmental impact

## Abstract

Over the last decade, poor diets and limited access to nutritious foods have been critical drivers of micronutrient deficiency in human health. However, food fortification at an industrialized scale in developed countries has helped eliminate deficiency-related diseases. In developing countries, fortified foods and biofortified materials have been delivered to nutrient-deficient communities. While these strategies have produced significant and acclaimed results, reports from the Food and Agricultural Organization suggest that over a quarter of the world’s population suffers from micronutrient deficiency. This implies that there are still declines in micronutrients in food products at different nodes along the food value chain (FVC). Hence, this study sets out to track micronutrient leakages at specific nodes of the FVC using potato household storage, processing, and consumption in the United States as a case study. The experiment was laid out in a full factorial design with three storage conditions (cupboard at (17.5–22.4 °C, 32.7–48% RH), refrigerator at (–1.8 – 0.89 °C, 37.5–66.1% RH) and ideal condition at (7.2–11.1 °C, 85.0–92.4% RH)), two storage times (2 weeks (±3 days) and 5 weeks (±3 days)) and three household processing pathways (boiling in water, baking at 204 °C, and frying in vegetable oil at 149–204 °C). Additionally, we explored the dynamics of optimal household storage and processing pathways by placing a high, low, or equal priority on environmental sustainability or nutrient retention. The results show that storing potatoes for 5 weeks (±3 days) and processing through boiling (in water at 100 °C), baking (at 204 °C), and frying (in vegetable oil at 149–204 °C) are associated with 33.5%, 40.3% and 15.0% greater nutrient loss than a similar processing scenario after 2 weeks (±3 days) of storage. Additionally, storing and processing potatoes after 5 weeks (±3 days) results in approximately 2.2 ± 0.7 times more damage to human health, ecosystem safety, and resource availability than storing and processing potatoes after 2 weeks (±3 days), averaged between the different storage conditions. Storing and processing after 5 weeks (±3 days) results in approximately 53.6 ± 10.3 times more damage to human health, species disappearing per year, and USD loss than freshly purchased and processed potatoes. Perhaps the most significant finding from the study is that storing potatoes in cupboards and boiling (BL-CP pathway) is optimal for achieving a sustainable healthy diet, as it yields the optimal combination of nutrient retention and low environmental damage. Insights from the study could be translated to support consumer decision-making as they weigh the value of environmental sustainability against nutrition in the context of household potato storage and processing.

## Introduction

The global food system, through its critical role in nutrition and economic development, has simultaneously impacted human health and the environment. In the developing world, malnutrition and lack of access to healthcare have led to millions of premature deaths^1,2^. In the developed world, consuming heavily processed foods has resulted in an elevation of several noncommunicable diseases, such as high cholesterol, diabetes, cancers, and cardiovascular disease, in record numbers^3^. In 2020, it was projected that 720 to 811 million people would experience hunger worldwide^4^. That same year, the COVID-19 pandemic exacerbated world hunger to 9.9% (a 1.5% increase) after remaining virtually unchanged in the previous five years^5,6^. Approximately 12% of the global population remains food insecure as of 2020^5,7^. Globally, malnutrition of all forms remains a challenge. These challenges are further intensified by environmental challenges such as climate change, which inevitably destroys our ecosystems^8–10^. Recent reports suggest that a shift to healthy diets that consider environmental sustainability can simultaneously reduce the food system’s human health impact and environmental burden^9,11^. However, the current food system has been designed not to promote human health or environmental sustainability but to increase crop yield and productivity. This approach has resulted in unintended consequences such as malnutrition (micronutrient deficiency), an important contributor to the global burden of diseases.

Fighting malnutrition requires multisectoral and multistakeholder interventions and coordination between researchers, funders, and policymakers. In developed countries, there is legislation and political support for food fortification at industrial levels, while in developing countries, large-scale food fortification and biofortification pathways have been designed to increase micronutrient availability^12–14^. Additionally, the EAT-Lancet Commission on healthy diets identified postharvest nutrient loss reduction as an important way to make food systems more efficient^15^. In analyzing common beans, Kwofie, et al.^16^ reported a general decline in mineral (calcium, iron, and zinc) content up to 88% at different value chain stages. In another study, Mba^17^ examined the effect of different household preprocessing techniques on the polyphenol content of beans. The results showed that increased hydration time and process water temperature significantly increased polyphenol degradation. For bean cultivars such as Msiska, 82% of the original polyphenol content of 144.2 GAE mg/g was lost in direct correlation with the rate of water uptake. In a follow-up study, Ellis, et al.^18^ found that within the common bean value chain, average product losses at postharvest handling, storage, and marketing were estimated to be 85.6%, 11.6%, and 2.8% of the total loss along the FVC in Zambia and Malawi, respectively. Furthermore, the study was extended to determine the economic and nutrient losses along the FVC. Food losses contributed to economic losses of $269,417.6 and $8,035.2 at the production and marketing stages, respectively. However, the true cost based on value-added loss estimations was $423,737.8 at the production stage. These studies highlight the significant impact of storage methods and household processing on the nutritional content available to consumers.

Home processing is ubiquitous worldwide and has many potential consumer benefits. However, many essential nutrients can be destroyed or removed depending on the processing pathway adopted. For example, phytochemicals and fiber are lost from peeling the outer layers of fruits, vegetables, and perhaps whole grains^19^. Additionally, the heating and drying of food can potentially destroy relevant vitamins and minerals. Despite nutrition intervention efforts, inadequate predictions of nutrient amounts in food could lead to the persistence of public health problems in nutritionally vulnerable populations. On the other hand, proper monitoring and predictions for nutrient dynamics would open the way to preventing nutritional losses, better investment guidance, and more effective national policies. Additionally, studies by Parajuli, et al.^20^, Mouron, et al.^21^ and Jungbluth, et al.^22^ showed that the consumer node (household processing and consumption) is a significant hotspot for most environmental impact categories within the FVC.

Building on the above studies and the gap highlighted, this study argues that attention should be given to food storage and processing at the household level, as it significantly contributes to micronutrient deficiency (i.e., hidden hunger) and environmental damage. First, as a case study, we tracked potato nutrient profile as it moved through the home processing timeline, from the retail to the table, to determine which interactions of storage conditions and processing factors have the most significant impact on the final nutritional content. Second, we estimated the environmental impact of each household storage and processing pathway through an environmental life cycle assessment (LCA) using the ReCiPe 2016 impact assessment method. Finally, a recommendation on the optimal storage and processing pathway was proposed by exploring the dynamics of placing high, low, and equal priority on nutrition and environmental sustainability using the Technique for Order of Preference by Similarity to the Ideal Solution (TOPSIS decision modeling). The priorities were set in harmony with sustainable development goals 2 (end all forms of malnutrition) and 12 (sustainable management and efficient use of natural resources), and the consumer desire for sustainability. Insights from the study could be translated to support consumer decision-making, as consumers weigh the value of environmental sustainability against nutrition in the context of household potato storage and processing. Additionally, with the COVID-19 pandemic spiking consumer interest in household processing of food for consumption, research under this theme could provide valuable insights to improve the nutrition and health of the population through the lens of sustainability. The entire structure of the study is as follows: the first section, including the introduction, begins by laying out the theoretical dimensions of the research and looks at how it could be adapted to every processed agricultural commodity for household consumption. The second section (Results and Discussion) draws together the study’s key findings to provide a well-rounded and detailed illustration through a case study approach. The third section (Conclusion) summarizes the main findings and recommendations of the study. The final section is concerned with the method employed for this study.

**Fig. 1:**
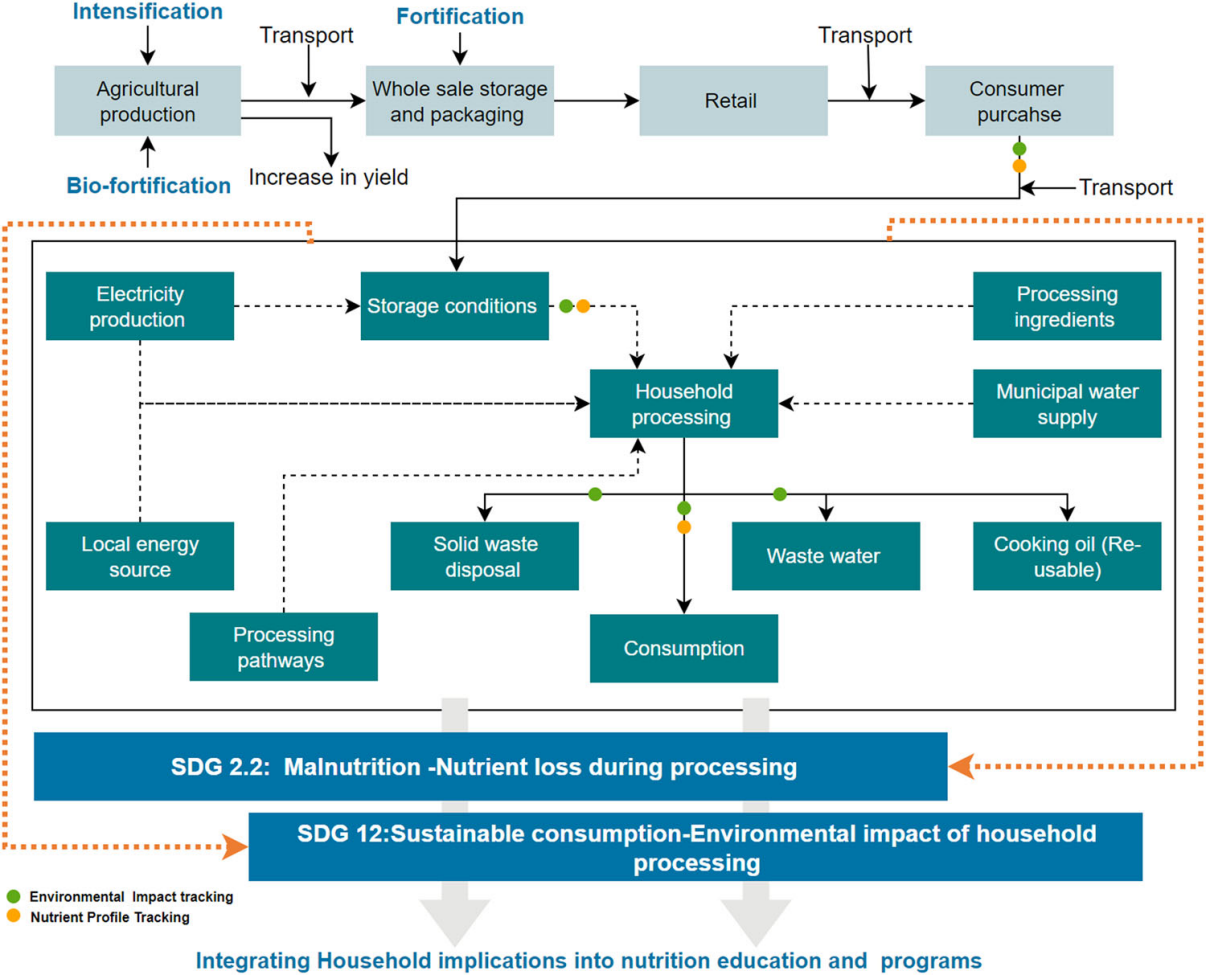
Theoretical framework to capture the nutrition, environmental and economic implications of household food processing.

Figure 1 presents the theoretical framework developed to analyze the nutritional and environmental impact of household storage and processing of potatoes. It maps the value chain from agricultural production to household storage, processing, and consumption. At the household value node, inputs include energy, processing pathways, ingredients, and municipal water supply. Food systems differ in size and structure from one country to another and between rural and urban areas, especially in developing countries. Hence, based on the contextual application of the framework and agricultural commodity of interest, various energy sources, water, and processing ingredients may be considered. The framework highlights the continuous attention given to addressing the multiple burdens of malnutrition along the value chain through agricultural intensification, biofortification, and fortification. In this study, the theoretical framework was applied to explore the sustainability burdens of household storage and processing of potatoes. The figure also indicates the UN Sustainable Development Goals (SDGs), which benefit from this research.

The environmental impact assessment expounds on the work of Parajuli, et al.^20^, who conducted an LCA of potato production and consumption in the United States. The study reported that the consumer value chain component contributes to approximately 47% of the total environmental impact; however, it failed to consider the nutritional and environmental impact at different storage and processing pathways at the household level. Again, several studies have concluded that a simple shift in consumers’ dietary patterns and behavior in developed countries while adopting sustainable processing pathways could reduce greenhouse gas emissions^3,23^. Recent surveys suggest that 65% of consumers seek sustainable products and services^24^. Another consumer report reveals a 23% (year-on-year) increase in sustainability-oriented food choices in 2020, with a 16% decline in health-oriented food choices^25^. Therefore, the proposed theoretical framework allows stakeholders and food system analysts to simultaneously track nutrient loss and the corresponding environmental implications of food to provide relevant consumer insights at the household level.

## Results and Discussion

### Effect of storage pathways on mineral composition

**Fig. 2:**
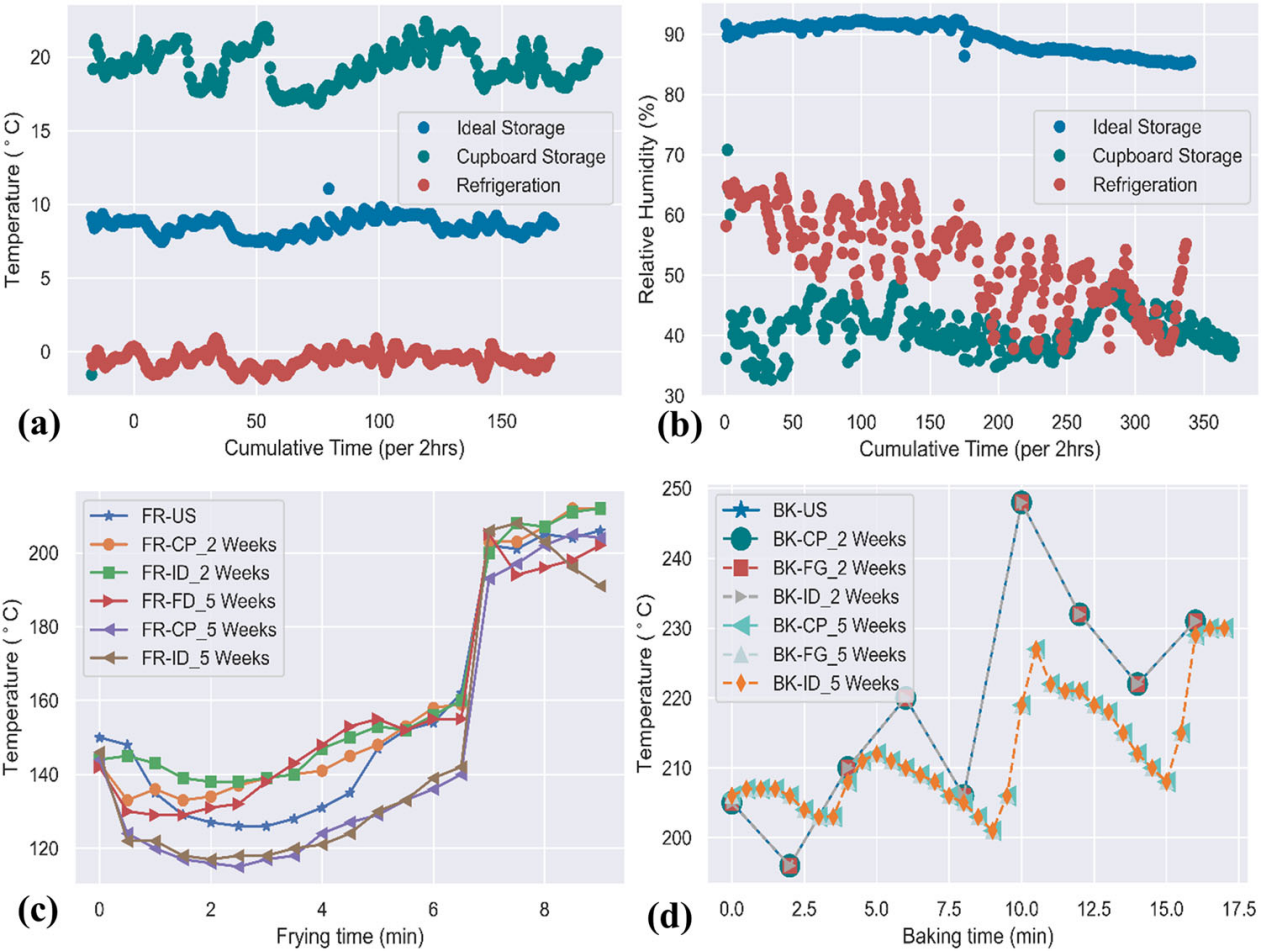
Temperature and humidity profiles for storage and household processing. **a** Temperature profile for the three storage conditions over 5 weeks (± 3 days). **b** Relative humidity profile for the three storage conditions over 5 weeks (± 3 days). **c** Temperature profile for frying processing and **d** temperature profile for baking.

Figure 2a, b show the temperature and relative humidity history fluctuations over the different storage periods modeled in this study. The temperature profiles for ideal storage (ID), refrigeration (FG), and storage in the cupboard (CP) from Fig. 2a were observed to range between (7.2–11.1 °C), (-1.8–0.89 °C), and (17.5–22.4 °C), respectively. Likewise, the relative humidity profiles from Fig. 2b ranged from 85.0 to 92.4%, 37.5 to 66.1%, and 32.7 to 48% for ideal conditions, refrigeration, and cupboard storage, respectively. The different storage conditions had implications on shrinkage in the potato samples. Stored potatoes lose weight by giving up water to the surrounding air through transpiration. Table 1 demonstrates the mineral content increase or degradation for raw (RW) samples under ideal conditions, refrigeration, and cupboard storage conditions for 2 weeks (±3 days) and 5 weeks (±3 days). The findings suggest a nonlinear relationship between storage time and mineral content, following trends in other studies^26,27^. For example, the sodium content decreased from 270 ± 4 ppm in the RW-US sample to 217 ± 1 ppm and 174 ± 0.3 ppm after storage in a refrigerator for 2 weeks (±3 days) and 5 weeks (±3 days), respectively, while other mineral contents, such as P and K, significantly increased from 1803 ± 1 ppm and 12159 ± 7 ppm to 3066 ± 10 ppm and 17917 ± 10 ppm after storage in the cupboard for 5 weeks (±3 days). Overall, storing in ideal conditions and cupboard over 2 weeks (±3 days) yielded lower mineral content, while storing for 5 weeks (±3 days) yielded a more significant amount than freshly purchased samples. While it was expected that there should be minimal mineral variation based on storage conditions, other studies have noted the significant spatial distribution of minerals in potato tubers. LeRiche, et al.^28^ reported higher P, Mg, K, S, Zn, and Cl concentrations at the center of potato tubers compared to the distal end. The Na, Fe, Al, and Si concentrations were greater at the distal ends and decreased toward the center of the potato tuber. In the same study, Cu and B showed no distribution pattern from the stem to the bud end. Singh, et al.^29^ also observed variations in the mineral concentration of potato tuber flesh (when peeled) and whole tubers. In this study, higher concentrations of all minerals except S were observed in the tuber flesh, while the highest mineral removal was observed for Fe (33.89%), followed by Cu (23.23%), Mg (20.77%), and Ca (19.94%). Thus, mineral variations between tubers near the surface compared to the cortex were suggested. Subramanian, et al.^30^ studied the three-dimensional distribution of mineral compositions from the surface to the inner flesh of potato samples. Again, in the tuber flesh, different minerals show distinct distribution patterns. For example, Mg on the surface layer of the tuber was measured to be 1.9 mg/g (dry basis); however, this mineral was measured to be 1.0 mg/g within the inner tissues (flesh) of the potato. Other minerals, such as P (3.4 mg/g), Ca (2.2 mg/g), and K (39.3 mg/g), were measured at the surface layers of the potato. However, within the fleshy regions, P, Ca, and K experienced 93%, 66%, and 90% reductions in mineral composition, respectively. Similarly, Sharma, et al.^31^ reported mineral losses between peeling and the flesh of potato varieties in India. The average mineral losses were estimated to be approximately 39.9%, 21.7%, and 15.4% and 12.9% and 39.9% for Fe, Mn, Cu, and Zn, respectively. The observed variations reported in the literature and the present study may be attributed to factors such as growth location, stage of development, soil type, soil pH, soil organic matter, fertilization, irrigation, and weather^32^. Other factors, such as soil mineral composition, mineral uptake rate in tubers, translocation of mineral elements to edible portions of tubers, bioavailability of minerals from plant tissues, and low mobility in phloem cells, could potentially result in the observed mineral variations. Additionally, genotypic variation imposes varying resistance and retention features on potatoes^33^. Another possible explanation for the variations in nutrients is the sprouting of potatoes, which was observed for all storage conditions by the end of the 5 weeks (±3 days) time condition. Sprouts were removed before processing and measuring, which could potentially have led to nutrient depletion. Sonnewald and Sonnewald^27^ reported a reduction in the nutrient quality of potatoes during storage due to sprouting. Nonetheless, the results presented here corroborate the current literature, where tuber mineral composition either increased or decreased during storage. Osunde and Orhevba^26^ observed an increase in Fe, Ca, Mg, Na, Ni, and Cu contents, while the Mn content was reduced after storing Pleurotus tuber regium for two and three weeks. Against the above observations, further research was conducted to investigate the inter-potato and surface-to-inner flesh variations in freshly purchased potato samples (see section 4 of the supplementary document) and those stored under three conditions (Cupboard (16.5 to 20.6 °C, 36.4-59.1% RH), Refrigeration (3.4 to 12.5 °C, 91-100% RH) and Ideal (2.4 to −2.1 °C, 60.6-94.3% RH)) for two weeks (see section 5 of the supplementary document). In this second batch of experiments, four Russet potato tubers were sampled and inter and intra-mineral variations were investigated. The results of this analysis are presented in Supplementary Table 5. The results in Supplementary Table 5 reveal wide variations for calcium (426 ± 1 ppm to 251 ± 5 ppm), phosphorus (6522 ± 20 ppm to 3062 ± 9 ppm), potassium (39,219 ± 50 ppm to 28,012 ± 60 ppm), and sodium (408 ± 10 ppm to 105 ± 4 ppm) for the four samples at the surface. In contrast, a lower mean concentration of macrominerals can be observed in the inner flesh region for sample potato tubers 1, 2, and 4 in Supplementary Table 5. Supplementary Tables 6 and 7 also show the mineral variations for storage under three conditions after two weeks. In both Supplementary Tables 6 and 7, we observed variations between the surface and inner parts of the different samples during storage, ranging from a 76% decrease in concentration (Na concentration in sample 6) to a −83% increase in concentration (P concentration in sample 3). When compared to the reference average of surface concentration of potato samples in Supplementary Table 5, we observe a 38% decrease in Ca concentration in sample 1- Cupboard (16.5 to 20.6 °C, 36.4-59.1% RH) and a 60% increase in Fe concentration in sample 3- Refrigeration (3.4 to 12.5 °C, 91-100% RH)). Additionally, in the inner flesh, a 47% decrease in Ca concentration in sample 4- refrigeration (3.4 to 12.5 °C, 91-100% RH)) and a 68% increase in Fe concentration in sample 1-cupboard (16.5 to 20.6 °C, 36.4-59.1% RH) is observed when compared to the average inner-part concentration in Supplementary Table 5. The results in Supplementary Tables 5, 6, and 7 support the current assertion of mineral variations at the inner and surface of potato tubers, which could consequently influence the changes observed during storage.

**Table 1:** Mineral degradation/increase during storage of potatoes under different conditions and times.

		2 WEEKS (±3 DAYS)			5 WEEKS (±3 DAYS)		
	RW-US*	RW-FG	RW-ID	RW-CP	RW-FG	RW-ID	RW-CP
Cal**	3668 ± 0.4	3656 ± 0.5	3630 ± 0.4	3689 ± 0.1	3565 ± 0.4	3628 ± 1	3607 ± 1
**Macrominerals (ppm)**							
Ca	489 ± 6	519 ± 3	543 ± 1	344 ± 2	478 ± 0.4	623 ± 2	527 ± 2
P	1803 ± 1	2709 ± 3	1603 ± 20	1627 ± 5	1967 ± 3	2511 ± 10	3066 ± 10
K	12159 ± 7	9382 ± 30	10595 ± 50	5506 ± 2	13273 ± 0.1	11879 ± 10	17917 ± 10
Na	270 ± 4	217 ± 1	136 ± 1	138 ± 1	174 ± 0.3	144 ± 1	144 ± 2
**Microminerals (ppm)**							
Al	9.5 ± 0.1	9.2 ± 0.1	8.8 ± 0.1	8.9 ± 0.1	7.9 ± 0.5	8.5 ± 0.1	7.5 ± 0.1
Cu	2.3 ± 0.1	2.1 ± 0.2	2.3 ± 0.1	<1.7	1.7 ± 0.1	2.3 ± 1	2.8 ± 0.1
Fe	10.7 ± 0.3	12.0 ± 0.2	6.3 ± 0.5	8.6 ± 0.5	7.3 ± 1	11.3 ± 1	11.7 ± 1
Mg	886 ± 0.1	709.5 ± 0.4	765 ± 3	656.5 ± 1	821 ± 2	1048 ± 2	1034 ± 3
Mn	15.2 ± 0.1	20.8 ± 1	14.7 ± 0.1	15.6 ± 0.1	14.8 ± 0.2	16.2 ± 0.2	15.0 ± 0.3
S	1278 ± 1	1139 ± 1	1080 ± 1	1150 ± 1	846 ± 1	1134 ± 3	1250 ± 2

*Note that: RW-US is Raw unstored sample; RW-FG is Raw Fridge samples; RW-ID is Raw Ideal Storage sample; RW-CP is Raw Cupboard; **Cal is calories measured in (Cal/gm ADB) ((For each storage condition, one potato tuber was randomly selected, and two samples were collected for duplicate analysis).

### Effects of sprouting on the safety of potato consumption

It is also important to highlight that if potatoes sprout during storage, they can contain potentially harmful levels of the Solanine toxin. Previous research indicates that storing potatoes at low temperatures in light can increase solanine concentrations, but depending on the levels consumed, they can be detrimental to human health^34^. Additionally, the presence of solanine is accompanied by an aversive bitter taste and noxious solanine gas^34^. Hence, it is expedient to consider solanine toxicity if potatoes are to be stored for an extended period in refrigerated conditions or have sprouted significantly. Therefore, sprouts should always be cut from sprouted potatoes before processing, and any green potato skin or flesh should likewise be cut out before processing.

### Effect of processing pathway on mineral composition

The choice of potato processing pathway was found to significantly affect the nutritional composition. Figure 2c, d show the temperature fluctuations for baking (BK) and frying (FR) treatments over the cooking period. The processing pathways resulted in a corresponding change in mass for the different storage scenarios. For the 2-week (±3 days) storage scenarios, mass decreases during frying and baking ranged between 40–45% and 27–33%, respectively. Similarly, the mass decrease for 5 weeks (±3 days) during frying and baking was observed to be 55–63% and 35–51%, respectively. The moisture evaporation from the surface of potato samples during frying and baking is due to simultaneous heat and mass transfer phenomena^35^. This finding is consistent with that of Sandhu and Takhar^36^, who reported faster structural degradation, moisture loss and oil uptake for higher frying temperatures.

**Fig. 3:**
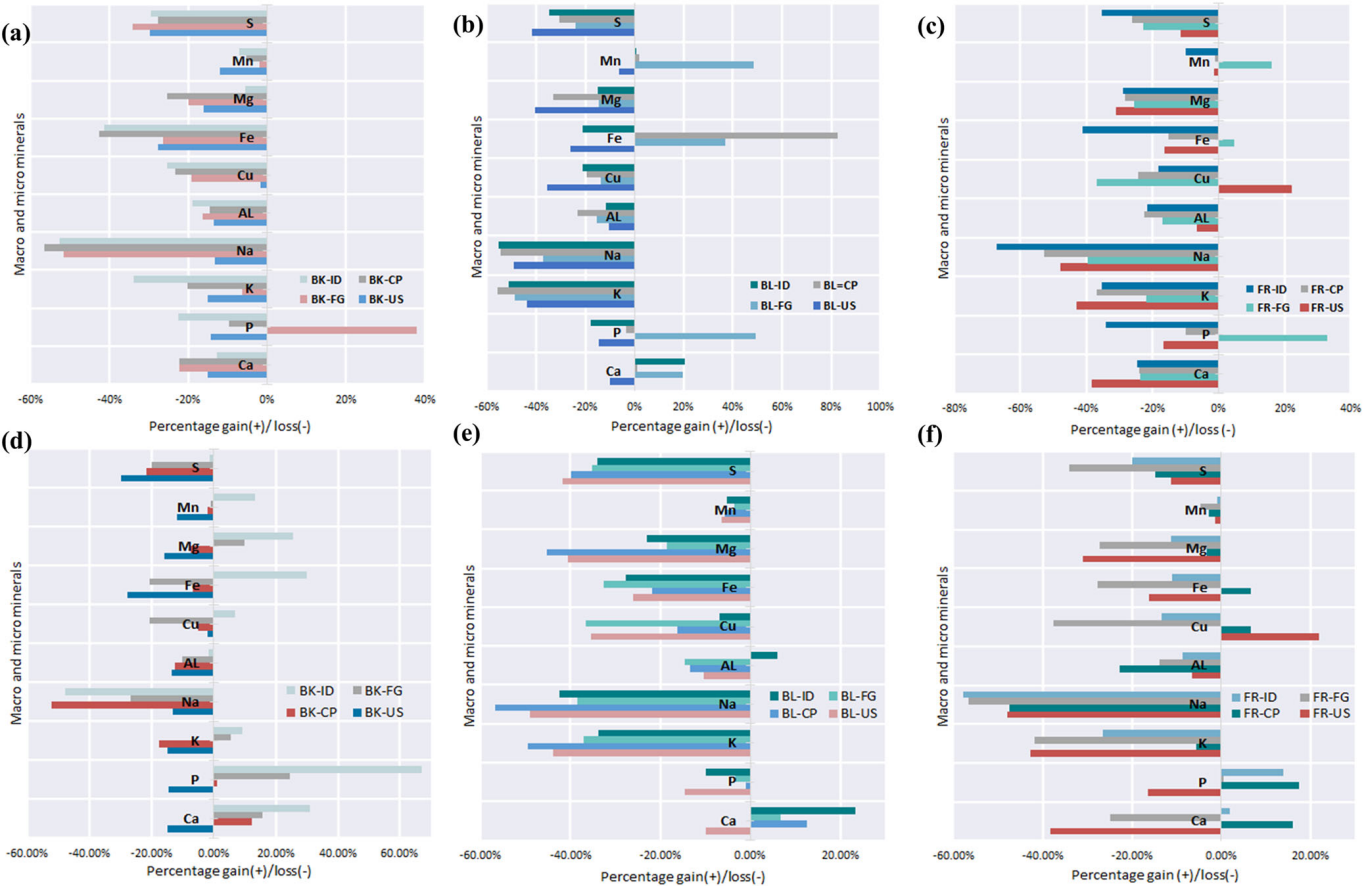
Nutrient variations during storage and processing scenarios. **a**–**c** Represent the nutrient reduction or gain for different processing and storage scenarios after 2 weeks (± 3 days); **d**–**f** represent the nutrient reduction or gain for different processing and storage after 5 weeks (± 3 days). Note: The meaning of the abbreviations is provided in the list of abbreviations and Fig. 4.

Conversely, there was an increase in mass content after boiling samples (BL), likely due to water absorption. For the case of 2 weeks (±3 days) storage conditions, the sample mass increased between 2 and 6%, while an increase of 8 and 22% was observed for 5 weeks (±3 days) storage conditions. It is also possible that the moisture loss due to storage conditions might have influenced the rate of water transfer during boiling. The results seem to suggest that longer storage time results in greater water absorption, although there were insufficient samples to draw a conclusion of statistical significance. Tables 2 and 3 present the mineral composition of baked and fried Russet potato samples after different storage conditions and times. Likewise, Supplementary Table 4 shows the mineral composition of boiled Russet potato samples under different storage conditions and times. Table 2 shows that among the different storage and processing scenarios, the baked-ideal conditions (BK-ID) pathway (5 weeks (±3 days)) resulted in the maximum mineral retention during consumption except for sodium. On the other hand, the baked-unstored (BK-US) pathway yields the maximum sodium level with a concentration of 2345 ppm. Additionally, the BK-ID pathway (5 weeks (±3 days)) resulted in an approximately 26% increase in mineral composition compared to the raw-unstored (RW-US) pathway. Figure 3d shows the mineral gain of the BK-ID pathway compared to other storage and processing pathways after 5 weeks (±3 days).

**Table 2:** Average mineral composition for baked potatoes after different storage conditions (2 samples were selected from each storage and processing condition. All analyses were done in duplicates).

	2 Weeks (± 3 days)				5 Weeks (± 3 days)		
	BK-US	BK-FG	BK-ID	BK-CP	BK-FG	BK-ID	BK-CP
Cal**	3669 ± 0.1	3732 ± 0.2	3697 ± 0.5	3714 ± 0.5	3637 ± 0.1	3646 ± 0.2	3622 ± 0.1
**Macro minerals (ppm)**							
Ca	416 ± 1	380 ± 2	427 ± 1	380 ± 0.2	566 ± 1	640 ± 1	549 ± 2
P	1544 ± 1	2486 ± 1	1396 ± 1	1629 ± 6	2247 ± 5	3011 ± 3	1820 ± 2
K	10345 ± 6	11396 ± 0.1	8064 ± 20	9706 ± 5	12822 ± 20	13275 ± 6	10033 ± 10
Na	235 ± 0.1	131 ± 0.0	129 ± 1	118 ± 1	198 ± 0.1	141 ± 1	130 ± 1
**Microminerals (ppm)**							
Al	8.2 ± 0.1	8.0 ± 0.2	7.7 ± 0.1	8.1 ± 0.4	8.5 ± 0.3	9.3 ± 0.1	8.3 ± 0.1
Cu	2.2 ± 0.1	1.8 ± 0.2	1.7 ± 0.1	1.7 ± 0.2	1.8 ± 0.1	2.4 ± 0.1	2.2 ± 0.1
Fe	7.7 ± 0.2	7.9 ± 0.4	6.3 ± 0.1	6.2 ± 0.2	8.5 ± 0.6	13.9 ± 0.3	10.0 ± 0.1
Mg	745 ± 1	711 ± 2	838 ± 0.2	662 ± 0.4	976 ± 3	1112 ± 1	822 ± 1
Mn	13.4 ± 0.1	14.9 ± 0.2	14.1 ± 0.0	14.4 ± 0.1	15.0 ± 0.1	17.2 ± 0.2	14.9 ± 0.0
S	899 ± 1	844 ± 3	903 ± 0.3	926 ± 2	1023 ± 3	1260 ± 3	1000 ± 2

**Cal is calories measured in (Cal/gm ADB). (For each storage condition, two potato tubers were sampled and processed by baking. After baking, two samples were collected for each storage-to-baking combination for duplicate analysis).

**Table 3:** Average mineral composition for fried potatoes under different storage conditions (2 samples were selected from each storage and processing condition. All analyses were done in duplicates).

	2 Weeks (± 3 days)				5 Weeks (± 3 days)		
	FR-US	FR-FG	FR-ID	FR-CP	FR-FG	FR-ID	FR-CP
Cal**	5143 ± 1	4555 ± 2	4744 ± 7	5055 ± 1	4499 ± 4	4761 ± 2	4369 ± 1
**Macro minerals (ppm)**							
Ca	302 ± 2	373 ± 1	369 ± 1	372 ± 4	368 ± 0.0	498 ± 1	567 ± 2
P	1505 ± 9	2395 ± 5	1188 ± 2	1623 ± 8	1814 ± 0.4	2052 ± 2	2119 ± 2
K	6964 ± 20	9509 ± 10	7864 ± 20	7671 ± 20	7079 ± 3	8932 ± 4	11483 ± 7
Na	141 ± 3	163 ± 3	89 ± 0.2	128 ± 2	117 ± 0.3	114 ± 1	142 ± 0.4
**Microminerals (ppm)**							
Al	8.9 ± 0.2	7.9 ± 0.4	7.5 ± 0.1	7.4 ± 0.1	8.2 ± 0.0	8.7 ± 0.5	7.3 ± 0.3
Cu	2.8 ± 0.2	1.4 ± 0.7	1.9 ± 0.3	1.7 ± 0.3	1.4 ± 0.0	2.0 ± 0.1	2.4 ± 0.2
Fe	9.0 ± 0.1	11.2 ± 0.4	6.3 ± 0.5	9.1 ± 0.8	7.7 ± 0.5	9.5 ± 0.8	11.4 ± 0.4
Mg	612 ± 4	660 ± 4	629 ± 3	636 ± 5	644 ± 4	786 ± 2	858 ± 2
Mn	15.0 ± 0.2	17.6 ± 0.4	13.7 ± 0.1	15.0 ± 0.2	14.5 ± 0.1	15.0 ± 0.2	14.8 ± 0.2
S	1133 ± 4	985 ± 1	828 ± 1	942 ± 4	844 ± 1	1022 ± 3	1088 ± 3

**Cal is calories measured in (Cal/gm ADB). (Two potato tubers were sampled from each storage condition and processed by frying. After frying, two samples were collected for each storage-to-frying combination for duplicate analysis.

In contrast, the pathways BK-ID and baked cupboard (BK-CP) for 2 weeks (±3 days) resulted in the lowest level of mineral content for consumption. Figure 3a shows that the mineral loss rates for the BK-ID and BK-CP pathways (2 weeks (±3 days)) were −24.8% and 24.7%, respectively.

Table 3 shows that the fried-cupboard (FR-CP) pathway (5 weeks (±3 days)) yielded the highest mineral content for calcium (16%), potassium (−5%), iron (7%), and magnesium (−3%) when compared to the raw-unstored (RW-US) pathway after processing. At the same time, the fried-refrigerator (FR-FG) pathway (2 weeks (±3 days)) resulted in the highest mineral retention for phosphorus (33%), sodium (−40%), and magnesium (16%). The observed increase in mineral content and retention may be due to the absorption of the frying oil and polar compounds released during oil degradation. The most significant mineral loss was observed for sodium and potassium in all storage and processing scenarios for 2 weeks (±3 days) and 5 weeks (±3 days) (Fig. 3). The results corroborate the work of Jayanty et al.^37^, who explored the influence of boiling, baking, microwaving, and frying on altering the anti-nutrient compounds and potassium content decrease (50% and 75% after boiling potato cubes and shredded tubers). Lachman, et al.^38^ reported that the peeling and cooking treatment of potatoes reduced phytochemical content; thus, boiling, compared to baking and microwaving, proved more favorable regarding phytochemical levels.

### Environmental impact analysis

**Fig. 4:**
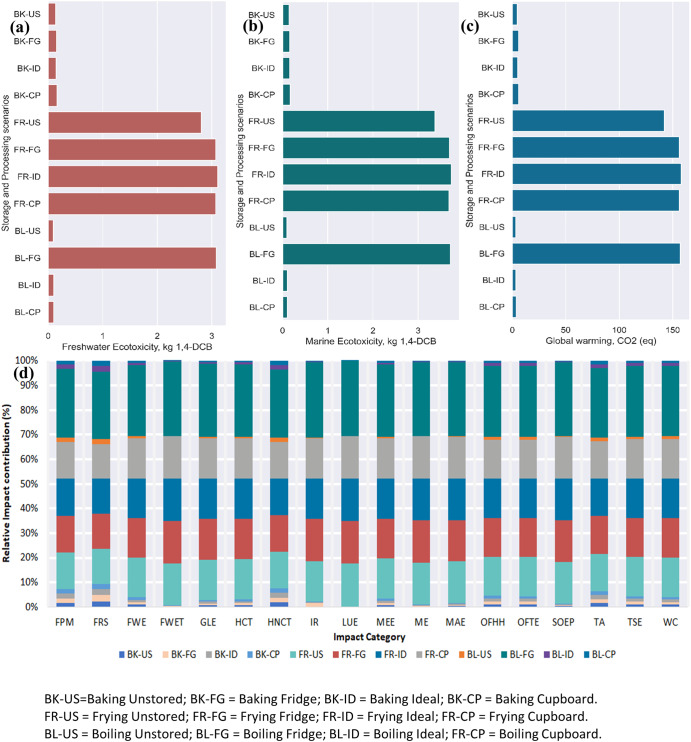
Midpoint impact assessment results for different storage and processing scenarios after 2 weeks (± 3 days). **a** Represents the characterized values for freshwater ecotoxicity as impact category, **b** impact value for marine ecotoxicity damage, **c** estimated global warming potential impact, and **d** relative impact contribution in percentage across all impact categories. The values presented are with respect to the functional unit. FPM Fine particulate matter formation, FRS Fossil resource scarcity, FEW Freshwater ecotoxicity, FET Freshwater eutrophication, GW Global warming, HCT Human carcinogenic toxicity, HNCT Human non-carcinogenic toxicity, IR Ionizing radiation, LU Land use, ME Marine ecotoxicity, MET Marine eutrophication, MRS Mineral resource scarcity, OFHH Ozone formation, Human health, OFTE Ozone formation, Terrestrial ecosystems, SOD Stratospheric ozone depletion, TAD Terrestrial acidification, TET Terrestrial ecotoxicity, WC Water consumption.

**Fig. 5:**
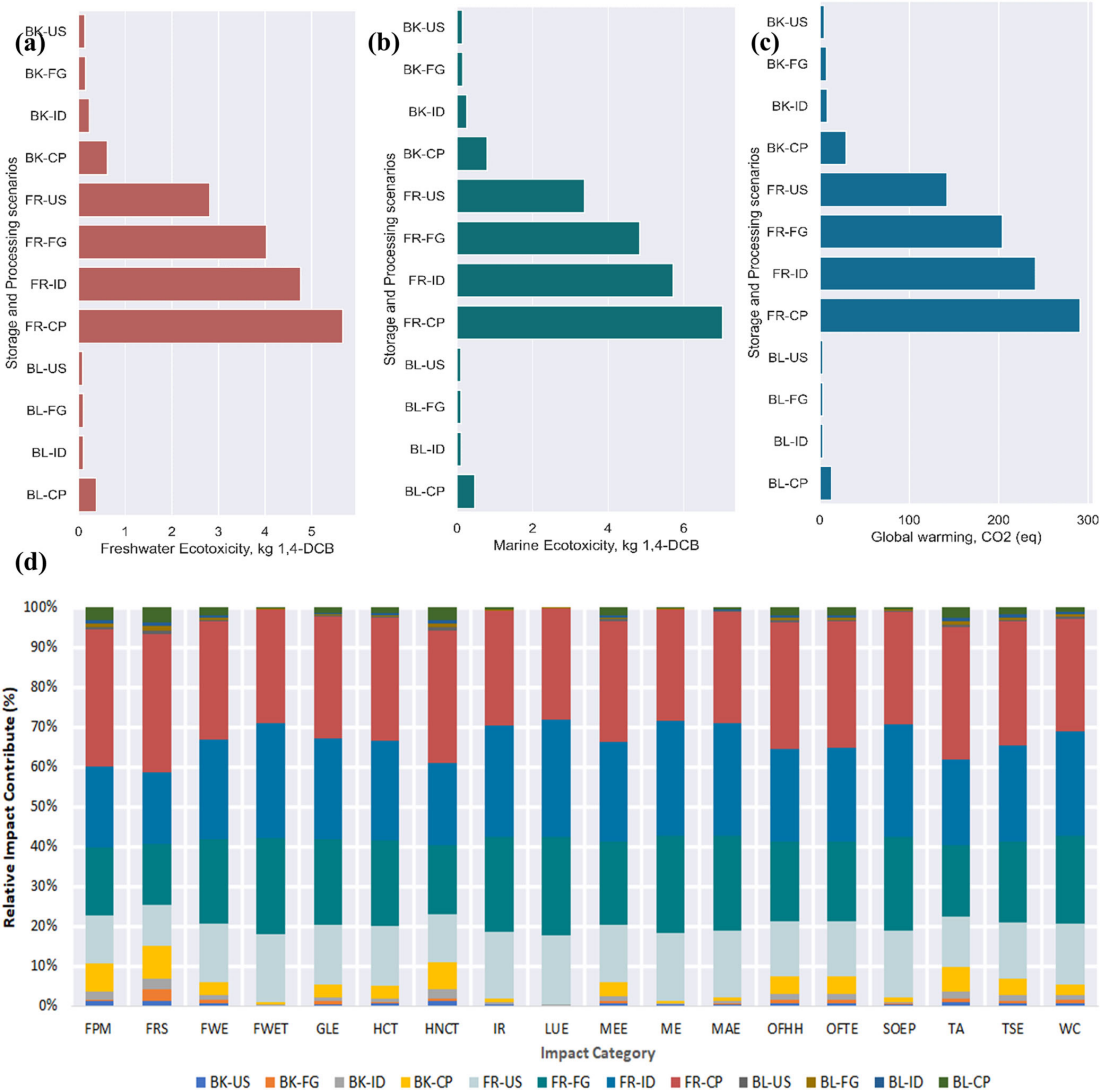
Midpoint impact assessment results (using ReCiPe 2016 Midpoint (H)) for different storage and processing scenarios after 5 weeks (± 3 days) of storage at different conditions. **a** Represents characterized values for freshwater ecotoxicity as impact category, **b** shows impact value for marine ecotoxicity, **c** displays the estimated global warming potential impact, and **d** presents the relative impact contribution in percentage across all impact categories. The values presented are with respect to the functional unit. FPM Fine particulate matter formation, FRS Fossil resource scarcity, FEW Freshwater ecotoxicity, FET Freshwater eutrophication, GW Global warming, HCT Human carcinogenic toxicity, HNCT Human non-carcinogenic toxicity, IR Ionizing radiation, LU Land use, ME Marine ecotoxicity, MET Marine eutrophication, MRS Mineral resource scarcity, OFHH Ozone formation, Human health, OFTE Ozone formation, Terrestrial ecosystems, SOD Stratospheric ozone depletion, TAD Terrestrial acidification, TET Terrestrial ecotoxicity, WC Water consumption. Note: The meaning of the abbreviations is provided in the list of abbreviations and Fig. 4.

Figures 4 and 5 show the environmental impact results caused by the different household storage and processing pathways for 2 weeks ( ± 3 days) and 5 weeks (±3 days). The impact categories with the highest normalized impact were freshwater ecotoxicity (Fig. 4a) and marine ecotoxicity (Fig. 4b), expressed as kilograms of 1,4-dichlorobenzene (kg 1,4-DCB (1,4-dichlorobenzene)) equivalents for a 100-year time horizon. Additionally, the characterized environmental impact results for global warming expressed as kg CO_2_ equivalence are presented in Fig. 4c. From Fig. 4a–c, it can be observed that storing and processing 1 kg of potato after 2 weeks (± 3 days) using the BL-FG pathway is associated with a toxicity impact equivalent to 0.53 and 0.64 kg 1,4-DCB for fresh and marine waters, respectively. Similarly, the boiled-fridge (BL-FG) pathway significantly impacts global warming, with an associated impact of 27.1 kg CO_2 (eq)_. Alternatively, the storage and processing scenario associated with the boiled-unstored (BL-US) pathway had the lowest environmental impact contribution. For a kg of processed potato, the BL-US pathway resulted in impacts of 9.16E-02 kg 1,4-DCB, 9.57E-02 kg 1,4-DCB, and 3.12 kg CO_2(eq)_ for freshwater, marine waters, and global warming, respectively. The environmental damage from the boiled-cupboard (BL-CP) pathway was an average of 97.3 ± 0.02% less than that from the BL-FG and fried-ideal (FR-ID) pathways. The highest environmental impact contribution was associated with the BL-FG (six) and FR-ID (eleven) impact categories. In contrast, the BL-CP pathway was associated with the lowest associated environmental impact across all impact categories.

All processing scenarios associated with the frying pathway resulted in a relatively high environmental impact compared to the BL-FG pathway. The extent of the environmental impact associated with the BL-FG pathway may be attributed to the water resources and electricity used in the storage and processing scenario.

Turning our attention to storing and processing scenarios after 5 weeks (±3 days), it can be observed from Fig. 5a–c that the FR-CP pathway resulted in the highest environmental impact contribution. The FR-CP pathway resulted in an associated impact equivalent to 5.67 kg 1,4-DCB, 7.03 kg 1,4-DCB and 2.91E + 02 kg CO_2 (eq)_ in freshwater, marine waters and global warming. The environmental impacts from the FR-CP pathway were more than 10 times greater than those from the BL-CP pathway for 2 weeks ( ± 3 days). Again, the BL-US pathway resulted in the lowest environmental impact release across 14 impact categories. At the same time, the baked-fridge (BK-FG) pathway showed the lowest environmental performance with associated impacts of 5.95E-03 kg PM2.5 eq, 1.31E-03 kg P eq, 1.65E + 00 kg 1,4-DCB and 1.06E-02 kg SO2 eq for the impact categories of fine particulate matter, freshwater eutrophication, human noncarcinogenic toxicity, and terrestrial acidification. By comparison, the lowest environmental release after 5 weeks ( ± 3 days) is approximately 3.5 times higher than the lowest impact release from the BK-CP pathway after 2 weeks ( ± 3 days). Again, the results in Fig. 5 are somewhat counterintuitive in the frying storage and processing pathways. We observe a steady increase for all impact categories from FR-US, fried-fridge (FR-FG), and FR-ID to FR-CP. For example, the FR-US, FR-FG, FR-ID, and FR-CP pathways were associated with equivalents of 2.81, 4.03, 4.76, and 5.67 kg 1,4-DCB in freshwaters, which corresponded to steady increases of 43%, 70% and 102%, respectively, with respect to the FR-US pathway. These findings were consistent with the work of Carvalho, et al.^39^, who reported greenhouse gas emissions (GHGE) of 1.4 kg CO_2 (eq)_ and 1.09 kg CO_2 (eq)_ per kg of homemade potato chips using a hot-air fryer and oil immersion deep frying. GHGEs were due to the use of natural gas and electricity as fuel sources. However, in our study, GHGE was relatively high due to the electricity used for storing and processing potatoes. Furthermore, this study was conducted in Brazil, which has different energy ratings than the United States. Other studies by Mouron, et al.^21^ and Ponsioen and Blonk^40^ reported 1.96 to 2.05 kg CO_2 (eq)_ per kg consuming potato fries with and without accounting for biowaste, respectively. These results indicate that consumers’ home processing choices can dramatically increase or decrease their environmental footprint. Supplementary Tables 2 and 3 present the midpoint results for all storage and processing scenarios for 2 weeks ( ± 3 days) and 5 weeks ( ± 3 days).

### Endpoint environmental implications

**Fig. 6:**
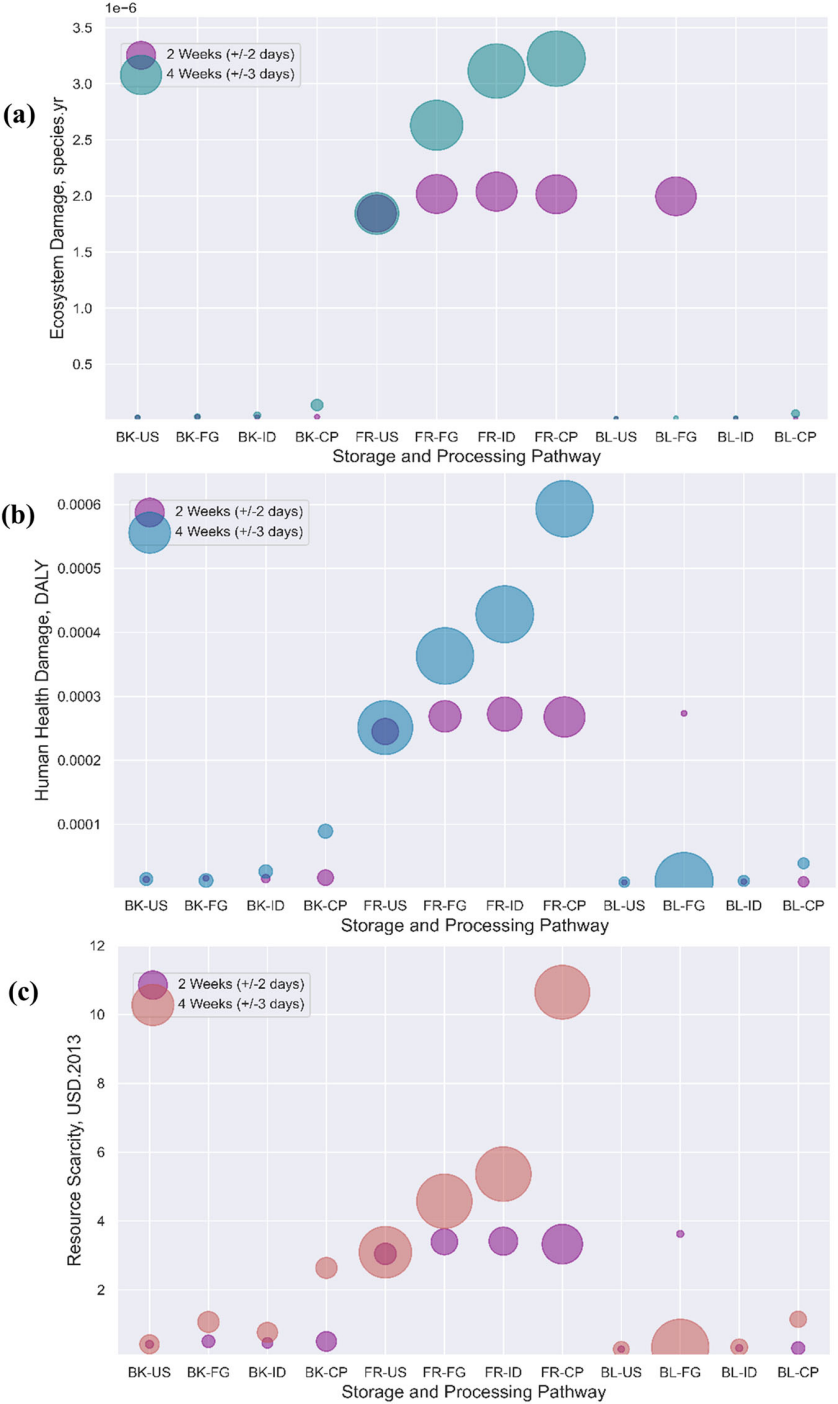
Endpoint areas of damage (using ReCiPe 2016 Endpoint (H)) comparison for the different storage and processing pathways (2 weeks (± 3 days) and 5 weeks (± 3 days)). **a** Shows the impact at the ecosystem endpoint damage level, b presents the impact on human health at the endpoint level, and **c** the impacts on resource availability potentially leading to resource scarcity. Note: The meaning of the abbreviations is provided in the list of abbreviations and Fig. 4.

After exploring the normalized and characterized midpoint results, the LCIA was further translated into endpoint areas of damage, and thus human health, ecosystem damage, and resource utilization. Figure 6 presents the final environmental impact scores for the damage to human health, ecosystem safety, and the availability of natural resources for the different household storage and processing scenarios. For the 2 weeks ( ± 3 days) storage and processing scenario, it was found that for 1 kg processed potato for consumption, the BL-FG pathway resulted in 2.73E-04 disability-adjusted life years, 2.00E-06 species disappeared per year and 3.62E + 00 USD loss for the endpoint categories. Similarly, in the case of 5 weeks ( ± 3 days), we observed that the FR-CP pathway resulted in a 5.93E-04 DALY, 3.22E-06 species disappeared per year and 1.06E + 01 USD loss for the respective three endpoint categories. Comparing the two case studies, processing after 5 weeks ( ± 3 days) results in approximately 2.23 times more damage to human health, ecosystem safety and resource availability than 2 weeks ( ± 3 days).

However, storing and processing after 5 weeks ( ± 3 days) using the FR_CP pathway results in approximately 64-, 186- and 38-fold damage to human health, species disappearing per year and USD loss compared to freshly purchased and processed potatoes. Thus, approximately 96.5 ± 9.8 times more damage to the endpoint areas of protection than freshly purchased and processed potatoes. Again, processing potatoes after 2 weeks ( ± 3 days) results in 29-, 117- and 13-times more damage to human health, species disappearing per year and USD loss compared to freshly purchased and processed potatoes. In summary, the results imply that storing potatoes for more extended periods at the household level has hidden environmental implications, which increase by a factor of two but are dependent on the type and time of storage.

### Uncertainty analysis

**Fig. 7:**
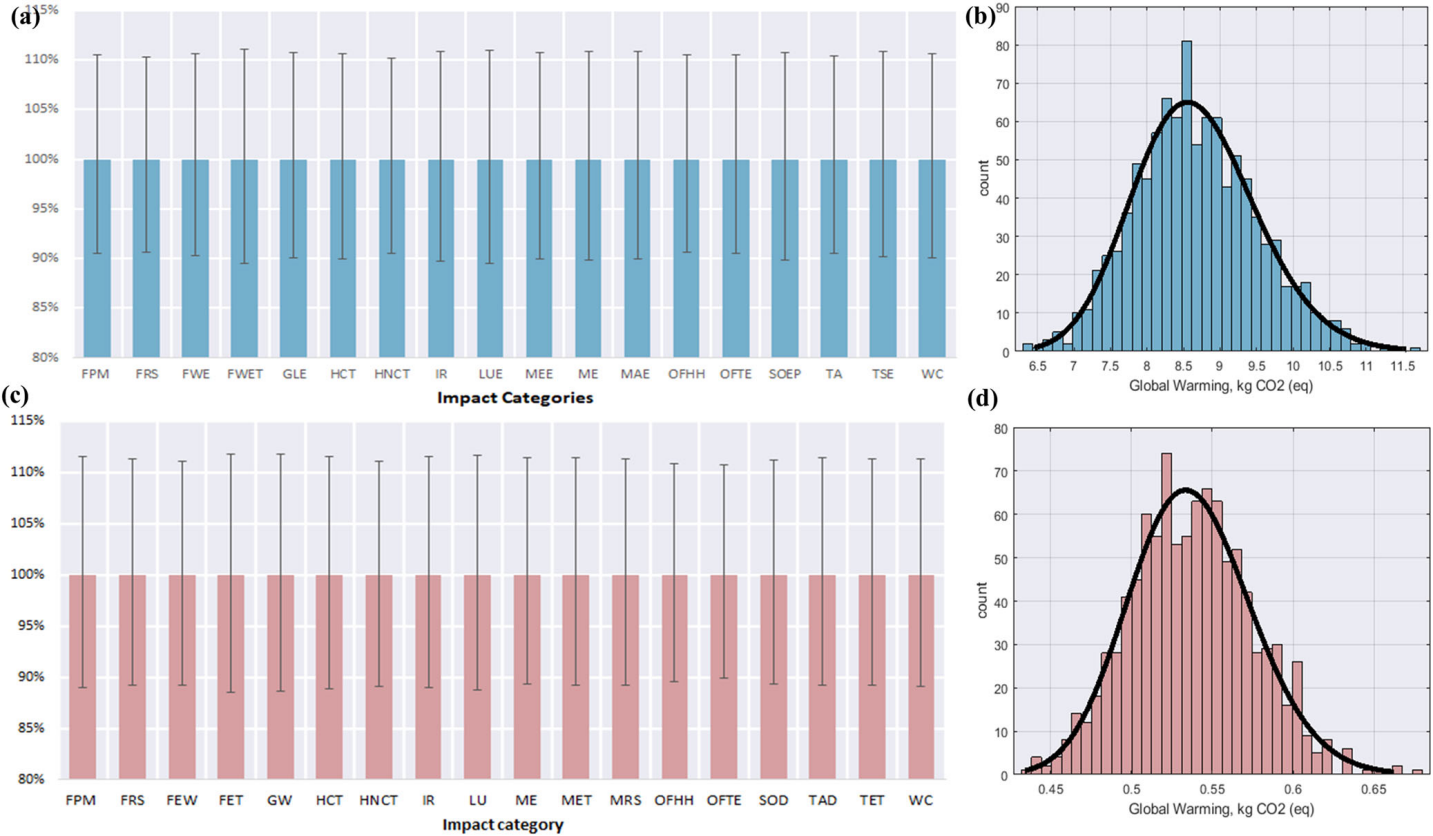
Uncertainty analysis for 1000 Monte Carlo iterations. **a** Shows the uncertainty for characterized LCIA profiles for BL-FG for 2 weeks (± 3 days) with error bars representing standard deviation from the mean value; **b** presents the probability distribution of characterized global warming potential (number of bin = 40) for BL-FG for 2 weeks (± 3 days); **c** the uncertainty for characterized LCIA profiles for FR-CP for 5 weeks (± 3 days) with error bars representing standard deviation from the mean value; **d** displays the probability distribution of characterized Global warming potential (number of bin = 40) for BL-FG for 2 weeks (± 3 days) and FR-CP for 5 weeks (± 3 days). FPM Fine particulate matter formation, FRS Fossil resource scarcity, FEW Freshwater ecotoxicity, FET Freshwater eutrophication, GW Global warming, HCT Human carcinogenic toxicity, HNCT Human non-carcinogenic toxicity, IR Ionizing radiation, LU Land use, ME Marine ecotoxicity, MET Marine eutrophication, MRS Mineral resource scarcity, OFHH Ozone formation, Human health, OFTE Ozone formation, Terrestrial ecosystems, SOD Stratospheric ozone depletion, TAD Terrestrial acidification, TET Terrestrial ecotoxicity, WC Water consumption. Note: The meaning of the abbreviations is provided in the list of abbreviations and Fig. 4.

Investigating the uncertainty associated with the LCA components facilitates improving the credibility and certainty of the LCA results. Therefore, based on the hypothesized probability distribution (Fig. 7b, d), this study computed the uncertainty ranges for the impact assessment profiles for the impact categories using Monte Carlo simulations. This was based on the hypothesized lognormal distribution and the data quality pedigree matrix in OpenLCA software. Table 4 presents the 95% confidence interval for all characterized LCIA results for FR-ID- 2 weeks ( ± 3 days) and FR-CP – 5 weeks ( ± 3 days) within the range (upper limits −95% and lower limits-5%). The coefficient of variation (CV) was computed for each characterized indicator. The CV measures the degree of dispersion of results from a characterized impact indicator. Thus, the lower the CV is, the lesser the dispersion and the greater our confidence in the LCA results. From Table 4, the CV values for FR-ID (2 weeks ( ± 3 days)) ranged between 5.87% and 6.66%, whereas the CV of FR-CP (5 weeks ( ± 3 days)) ranged between 6.35% and 7.08%. The error bars in Fig. 7a, c represent the uncertainty range in terms of the ratio of the 5^th^ and 95^th^ percentiles of the upper and lower limits to the mean impact scores. The error bars in Fig. 7(a) ranged between 9.39% and 11.06%, while those in Fig. 7c ranged between 10.1% and 11.9%. Comparatively, the error bars indicate that a more considerable degree of uncertainty is introduced into land impact, water resource and toxicity impact scores due to relatively considerable uncertainty in freshwater and marine eutrophication, marine ecotoxicity and water consumption for the case of FR-ID (2 weeks ( ± 3 days)).

**Table 4:** Uncertainties for characterized LCIA profiles for storage and processing scenarios with the most significant impact.

Impact categories	FR-ID- 2 weeks (± 3 days)					FR-CP – 5 weeks (± 3 days)				
	Mean	SD	CV %	LL	UL	Mean	SD	CV %	LL	UL
FPM	2.21E-03	1.33E-04	6.01%	2.00E-03	2.45E-03	4.51E-04	3.14E-05	6.97	4.02E-04	5.04E-04
FRS	1.69E-01	9.94E-03	5.87%	1.53E-01	1.87E-01	3.07E-02	2.11E-03	6.89	2.74E-02	3.42E-02
FEW	4.96E-02	3.06E-03	6.17%	4.48E-02	5.49E-02	1.31E-02	8.89E-04	6.76	1.17E-02	1.46E-02
FET	6.56E-03	4.37E-04	6.66%	5.87E-03	7.29E-03	1.76E-03	1.25E-04	7.08	1.56E-03	1.97E-03
GW	2.16E + 00	1.38E-01	6.41%	1.94E + 00	2.39E + 00	5.38E-01	3.79E-02	7.04	4.77E-01	6.01E-01
HCT	7.44E-02	4.73E-03	6.36%	6.69E-02	8.23E-02	1.86E-02	1.30E-03	6.98	1.65E-02	2.07E-02
HNCT	4.85E-01	2.88E-02	5.94%	4.39E-01	5.34E-01	1.11E-01	7.54E-03	6.77	9.93E-02	1.24E-01
IR	2.92E-02	1.90E-03	6.51%	2.62E-02	3.24E-02	7.91E-03	5.49E-04	6.94	7.04E-03	8.82E-03
LU	1.79E + 00	1.19E-01	6.64%	1.60E + 00	1.98E + 00	4.92E-01	3.44E-02	7.00	4.36E-01	5.49E-01
ME	5.42E-02	3.38E-03	6.24%	4.88E-02	6.00E-02	1.37E-02	9.39E-04	6.85	1.22E-02	1.53E-02
MET	1.36E-03	8.73E-05	6.44%	1.22E-03	1.50E-03	3.78E-04	2.58E-05	6.83	3.37E-04	4.21E-04
MRS	3.43E-03	2.18E-04	6.37%	3.09E-03	3.80E-03	9.54E-04	6.48E-05	6.80	8.51E-04	1.06E-03
OFHH	2.84E-03	1.71E-04	6.02%	2.58E-03	3.14E-03	7.32E-04	4.76E-05	6.50	6.56E-04	8.13E-04
OFTE	3.15E-03	1.88E-04	5.97%	2.85E-03	3.48E-03	8.44E-04	5.36E-05	6.35	7.59E-04	9.35E-04
SOD	4.88E-06	3.12E-07	6.38%	4.39E-06	5.41E-06	1.36E-06	9.18E-08	6.77	1.21E-06	1.51E-06
TAD	3.17E-03	1.90E-04	6.00%	2.87E-03	3.50E-03	7.10E-04	4.83E-05	6.81	6.33E-04	7.91E-04
TET	1.69E + 00	1.04E-01	6.14%	1.52E + 00	1.87E + 00	4.23E-01	2.87E-02	6.79	3.77E-01	4.70E-01
WC	6.37E-02	3.99E-03	6.26%	5.73E-02	7.05E-02	1.80E-02	1.24E-03	6.87	1.61E-02	2.01E-02

*FPM* Fine particulate matter formation, *FRS* Fossil resource scarcity, *FEW* Freshwater ecotoxicity, *FET* Freshwater eutrophication, *GW* Global warming, *HCT* Human carcinogenic toxicity, *HNCT* Human non-carcinogenic toxicity, *IR* Ionizing radiation, *LU* Land use, *ME* Marine ecotoxicity, *MET* Marine eutrophication, *MRS* Mineral resource scarcity, *OFHH* Ozone formation, Human health, *OFTE* Ozone formation, Terrestrial ecosystems, *SOD* Stratospheric ozone depletion, *TAD* Terrestrial acidification, *TET* Terrestrial ecotoxicity, *WC* Water consumption.

### Trade-off analysis

**Fig. 8:**
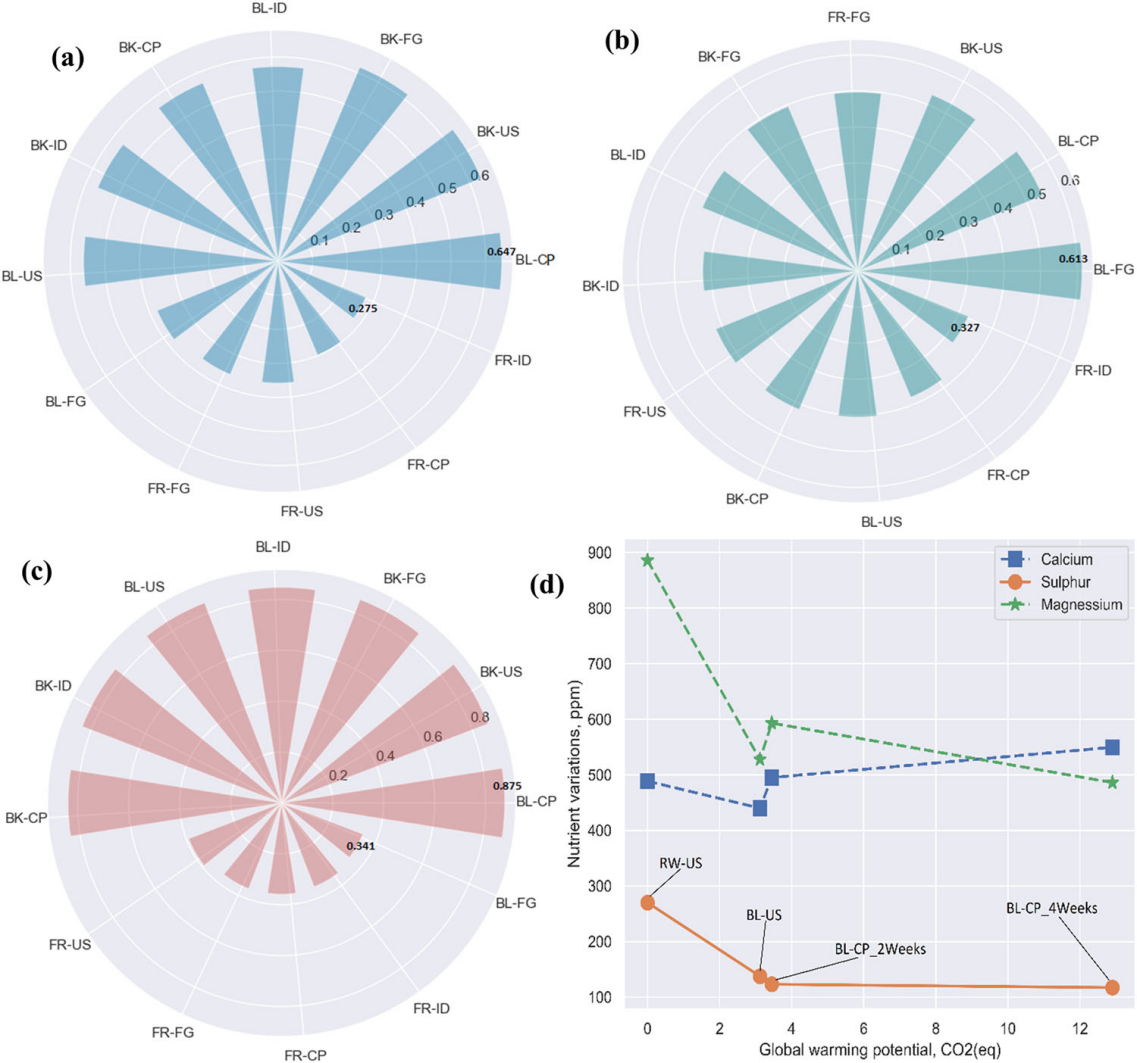
Contextual variations in the optimal household storage and processing after 2 weeks (± 3 days). **a** Equal importance placed on the environment and nutrition, **b** nutrition a higher priority than the environment and **c** environmental sustainability a higher priority than nutrition; **d** nutrient loss/gain against global warming potential (ReCiPe 2016 Midpoint impact assessment).

A recent consumer report by the International Food Information Council 2021 Food & Health Survey (IFIC 2021) suggests that almost 60% of consumers recognize the need for food products they purchase or consume to be environmentally sustainable, an increase from 54% in 2019^24^. In addition, another Global Sustainability Study conducted by Simon-Kucher and Partners revealed a significant global paradigm shift in how consumers view sustainability and have developed an increased willingness to pay more for sustainable products and services^41^. Based on this premise, this section explores the dynamics of determining the optimal household storage and processing pathway by examining how the optimal home potato processing method differs when different priority levels are placed on environmental sustainability and nutrient content during food consumption. Figure 8 presents the results of implementing TOPSIS decision modeling under different consumer priorities after 2 weeks ( ± 3 days). For brevity, a similar analysis on 5 weeks ( ± 3 days) storage and processing scenarios was not investigated. Figure 8a–c demonstrate the variation in performance score and optimal household storage and processing pathways considering three consumer priority ratings. This methodology allows researchers to evaluate best practices and deliver recommendations based on consumers’ priorities. A practical significance threshold of 1% was set according to the recommendations of Kruschke^42^ and Agyemang, et al.^12^ to compare the performance scores of two household storage and processing pathways. In other words, two storage and processing pathways were considered practical equivalents if their difference in performance score lies within [−1%,1%].

### Context of environmental importance

In the context of potato storage and consumption at home with a high priority on environmental sustainability, it can be observed from Fig. 8c that BL-CP, BK-US, and BK-FG ranked first, second and third, with performance scores of 0.875, 0.861 and 0.857 after 2 weeks ( ± 3 days). The closeness in performance score and practical insignificance of 1% between BL-CP and BK-US imply that consumers who prioritize environmental sustainability can adopt both storage and processing pathways. Additionally, the BL-CP and BK-US pathways were associated with approximately 13% and 25% nutrient loss, respectively, whereas BL-CP resulted in 36% less environmental impact than BK-US. Hence, for consumers with a high priority for environmental sustainability, storing and processing potatoes using the BL-CP pathway will reduce the environmental impact while still providing substantial nutritional value.

### Context of nutritional importance

In the context of nutritional gains being a high priority to the consumer, Fig. 8b shows that BL-FG, BL-CP, and BK-US ranked first, second and third with performance scores of 0.61, 0.55, and 0.53, respectively. A significant difference of 11% and 14% is observed between (BL-FG and BL-CP) and (BL-FG and BK-US), indicating practical significance in the processing pathways to adopt. Additionally, BL-FG was associated with a 13% and 25% lower nutritional loss than BL-CP and BK-US, respectively. In terms of calories supplied, BL-FG provides 3732 ± 0.2 Cal/mg ADB, while BL-CP and BK-US contain 3697 ± 0.3 and 3668 ± 0.4 cal/mg ADB, respectively. Nevertheless, BL-FG is associated with a 48 ± 10 and 36 ± 8 times greater environmental impact than BL-CP and BK-US.

### Context of equal importance

Figure 8a shows the ranking and performance scores of the different pathways in the context of placing equal importance on nutrition and environmental sustainability. The results demonstrate that the BL-CP, BK-US, and BK-FG pathways ranked first, second, and third, respectively, with performance scores of 0.65, 0.64, and 0.62. A practical significance of 1.6% and 4.6% is observed between (BL-CP and BK-US) and (BL-CP and BK-FG). Unlike the context of environmental sustainability priority, here, the BL-CP pathway and BK-US cannot be adopted simultaneously. Overall, storing and processing potatoes through the BL-CP pathway after 2 weeks ( ± 3 days) is associated with lower environmental impact and provides relatively beneficial values for nutrients and calories compared to other pathways explored in this study.

### Implications of the study

With the advent of global climate change impacts, which are characterized by temperature increases, drought and shifts in rainfall, the importance of sustainable healthy diets has become more critical. The Global Panel on Agriculture and Nutrition, through its first report in 2016, raised the attention of policymakers and stakeholders to the health and nutritional implications of food consumption. The subsequent report in 2021 drew attention to the environmental impact of food consumption. However, policymakers alone cannot bear the responsibility of turning global challenges around. Hence, the results of this study imply that individuals have a critical role to play, as their household storage and processing choices could significantly delineate potential global challenges around climate change and malnutrition. Additionally, adopting the theoretical framework for simultaneous nutrient leakage and environmental impact tracking could be applied to different agricultural commodities to enlighten and nudge consumers toward a more sustainable and healthier lifestyle. This could significantly reduce malnutrition through sustainable household food processing as we strive to achieve full net zero impact within the food system. Furthermore, applying the theoretical framework to different agricultural commodities could inform international bodies, donor agencies and nutrition intervention designers on household food storage and processing dynamics and their respective influence on nutritional content for target communities in Africa, Southern Asia and Latin America.

This study evaluated the nutritional variation and associated environmental impact during household potato storage and processing through an experiment simulating common household processing pathways. The results indicate that fried potatoes that have been stored in the cupboard for 5 weeks ( ± 3 days) have approximately 97 ± 10 times more damage to human health (DALYs), ecosystems (species-years), and resource availability (USD) than freshly purchased and processed potatoes. Similarly, boiling potatoes stored in the refrigerator after 2 weeks ( ± 3 days) causes approximately 54 ± 10 times more damage to human health (DALYs), ecosystems (species-years), and resource availability (USD) than freshly purchased and processed potatoes. From a nutritional perspective, storing and processing potatoes after 2 weeks ( ± 3 days) is associated with 56% and 67% nutrient loss for boiling, baking, and frying pathways, respectively. However, nutrient loss decreases after 5 weeks ( ± 3 days) of storage and processing, with 22%, 16%, and 56% nutrient loss for boiling, baking, and frying, respectively, compared to freshly purchased and unprocessed potatoes. The findings suggest that storing potatoes for an extended period has more significant environmental impacts, but the nutritional benefits may sometimes be inconclusive due to variations in mineral distribution within potatoes. However, if we consider environmental sustainability a key priority to consumers, potatoes stored and processed after 2 weeks ( ± 3 days) through boiling and the cupboard pathway (BL-CP) will be an optimal approach. If nutrition was a high priority, potatoes stored in the fridge and boiled (BL-FG) pathway would be optimal. In summary, storing potatoes in cupboards and processing them through boiling will support achieving a sustainable healthy diet. While this study does not provide a holistic understanding of nutrient leakage across the entire value chain, the findings contribute to understanding nutrient leakages and losses from production to consumption.

## Methods

### Method Framework

**Fig. 9:**
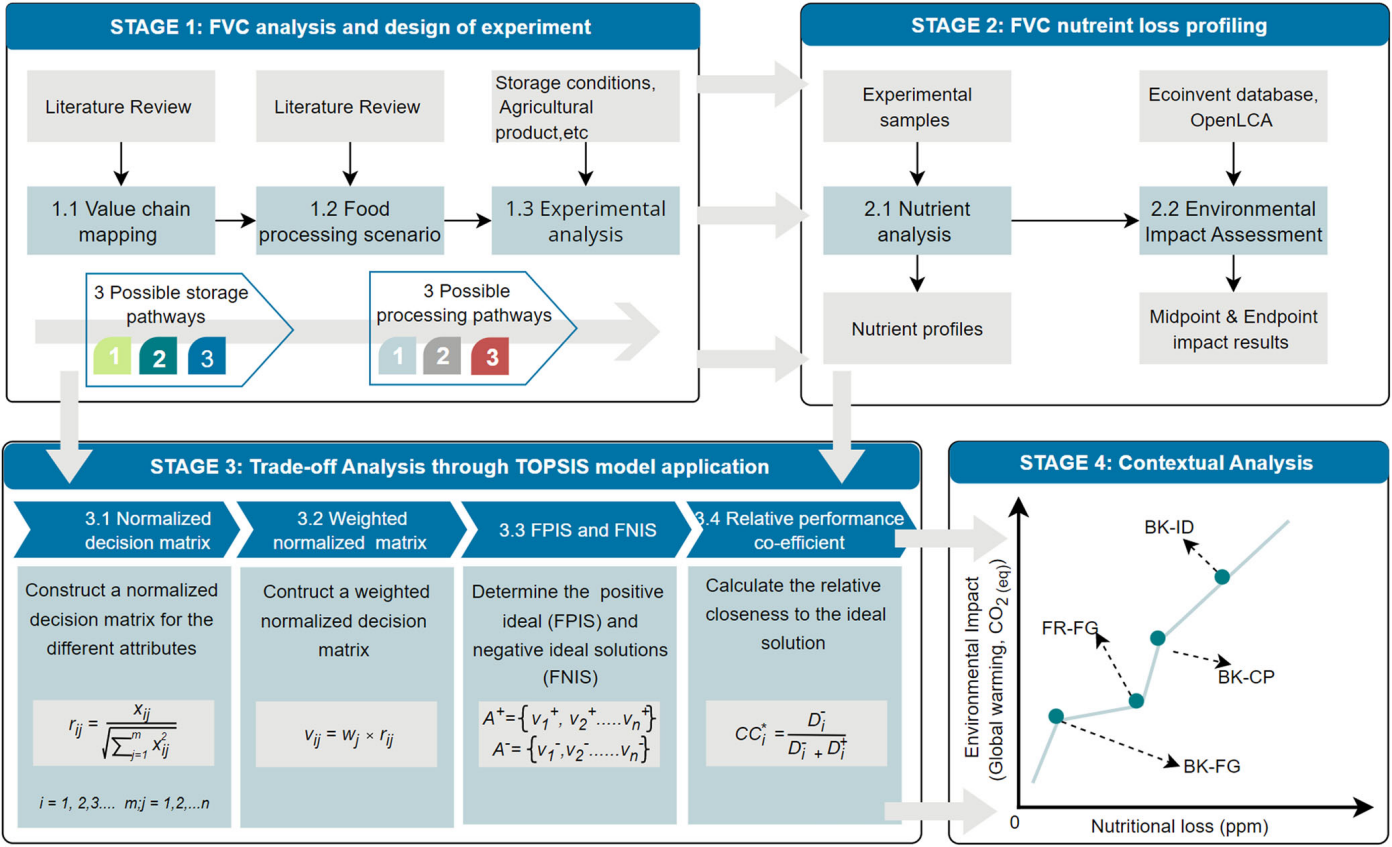
Methodological framework for the study.

Figure 9 shows the method framework developed from the theoretical framework presented in Fig. 1. From Fig. 9, four main stages were employed to deliver the study’s objective. The first stage involved value chain mapping, analysis, and experimental design. A literature review was conducted to determine potato value chain components, household storage practices and processing scenarios. The literature review was complemented by a household survey to ascertain the different storage and processing pathways often employed. From here, three household storage practices were determined: storage in cupboards at 21 °C, refrigeration at -0.5 °C and an ideal (8.9 °C, with 90% humidity)^43^. Similarly, three household processing pathways were identified: frying, boiling and baking. Thus, household storage conditions, storage duration and processing pathways were factors significantly driving nutritional variations in Russet potatoes’ nutrient availability during consumption. Building on the above, a factorial experimental design was used to determine the effects of these factors at the level of nutrition and environmental sustainability. The experiment was conducted over 5 weeks ( ± 3 days). In stage two, samples from the experiment were taken at different time intervals, processed and subjected to nutrient profiling. Additionally, an environmental LCA of each storage and processing pathway was modeled and assessed. In stage three, we leveraged the TOPSIS decision model to explore the dynamics of determining the optimal household storage and processing pathways. Stage four built on the results from stage three to examine how placing different levels of priority on environmental sustainability and nutrient availability could influence the choice of storage and processing pathway to adopt. It seeks to model current consumer trends and behavior toward environmental sustainability and investigates the possible trades-off to deliver a sustainable and healthy diet culture at the household level.

### Potato value chain

Over the past century, potatoes have been a staple vegetable in the American diet. In 2021, approximately 409.6 million cwt of potatoes were produced in the United States. As of 2019, the economic value of potatoes was estimated to be $3.94 billion, with an average market price of $9.84 per cwt^44^. Approximately 121.3 lb/capita of fresh and processed potatoes are consumed annually. From a market share perspective, potatoes are consumed in various forms. The most popular processed forms are frozen (34%), chipped (12%), dehydrated (10%), or canned (1%). In addition, 28% of potatoes produced are consumed fresh, while 15% are used as seeds. Research has shown that potatoes, even in processed forms, can significantly contribute to meeting the nutritional requirements of children and adolescents. For example, it contributes 10% of daily fiber, vitamin B6, and potassium and 5% of thiamine, niacin, vitamin C, vitamin E, vitamin K, phosphorus and copper^45^.

A typical potato value chain comprises three central components: production, processing, and consumption. The production component captures related raw materials such as fuel, agrochemicals, water, and machinery. Activities such as harvesting and packaging occur between production and processing, which include primary, secondary (corrugated boxes) and tertiary (pellet) materials of the freshly harvested potato. Freshly packaged potatoes are sent to retail and process centers before consumer purchase, household processing, and consumption. Parajuli, et al.^20^ present a detailed description of the entire value chain.

### Simulating household purchase and storage

Four bags (5 lb each) of Russet potatoes were purchased from a Walmart retail shop to simulate consumer purchases of potatoes from retail. Walmart was chosen based on the results of a preliminary self-conducted survey. The potatoes were placed in three simulated household storage conditions. Temperature and relative humidity readings for each storage condition were measured with Kestrel Drop sensors. The three storage conditions were (a) cupboard, in which an open container was placed in a dark cabinet with a central heating set at an average temperature of 20 °C and 30-45% relative humidity, (b) refrigeration, a storage condition where the open container was placed in a refrigerator with the temperature setting on medium (corresponding to an average of -0.5 °C and 40-60% relative humidity), and (c) ideal conditions, in which a closed container containing potatoes and a potassium chloride salt solution (to maintain high humidity) were placed in a refrigerator set at 8.9 °C. The average relative humidity level was 90%. The respective temperatures were selected following the recommendations of^43^ for ideal storage conditions and the common consumer storage methods indicated in the self-conducted survey.

### Simulating household processing and sampling

Before storage, fresh potato samples were processed under the three household processing strategies. Then, after 2 weeks (± 3 days) and 5 weeks (± 3 days), samples were taken and subjected to similar processing strategies. Again, 2 weeks (± 3 days) and 5 weeks (± 3 days) were selected based on an initial consumer survey of the time of purchase to process potatoes. The processing conditions modeled include (a) frying; samples were cut into circular cross sections ¼ inch in thickness using canola oil as the cooking medium at 149° F for 7 minutes, then removed and fried at 204 °F for two more minutes. (b) Baking: samples were cut into circular cross sections ¼ inch in thickness and placed into a preheated 204 °C oven for 17 minutes, and (c) Boiling: samples were cut into circular cross sections ¼ inch thick and placed into boiling water for 15 minutes. This was adapted from Bittman^46^ and Rombauer, et al.^47^. The processing temperatures were measured using a Taylor brand temperature probe in the cooking vessel.

After processing, samples from each storage and their corresponding processing pathways were collected and analyzed. Finally, the nutrient profile of each processed sample was compared to a raw/uncooked sample. Supplementary Table 1 presents the experimental variables for the complete factorial design. This full factorial was created using JMP software, which details the different combinations of factors and levels. The dependent variables in the experiment were nutritional content and environmental impact. A significant benefit of the full factorial design is that it accounts for all combinations of factors. Table 5 presents a combination of experimental variables for a complete factorial design and the independent variables (time, storage condition, and cooking method). The levels for each factor are as follows:

**Table 5:** Factors measured during the experiment.

S/N	Variable	Factor level
1	Storage time	Unstored (US)
		17 days (approximated to 2 weeks (± 3 days))
		33 days (approximated to 5 weeks (± 3 days))
2	Storage condition	Ideal (48 °F, 90% relative humidity, the closest approximation of conditions presented in^57^) (ID)
		Refrigerator (average 31 °F, 40-60% relative humidity) (FG)
		Cupboard (average 68 °F, 30-45% relative humidity) (CP)
3	Processing method	Raw/uncooked (RW)
		Baked (BK)
		Boiled (BL)
		Fried (FR)

### Nutritional analysis

The nutritional analysis of the mineral, fat and caloric content of the samples was performed by the University of Arkansas Central Analytical Lab according to the following protocols:

- Fat content: The fat content was determined using the AOCS AM 5-04 method of fat extraction, which uses petroleum ether to remove triglycerides from the sample.
- Caloric content: The calorie content was measured using a Parr 6200 Automatic Adiabatic bomb calorimeter.
- Complete Mineral Analysis: The mineral analysis was performed by digesting a 0.25 g dried powdered sample with 3 ml nitric acid and 1 ml hydrogen peroxide and then analyzing the resulting mixture using an ICP‒OES spectrometer.

The dry matter content was also determined by drying a 2 g potato sample in a 110 °C oven for 12 h. Finally, the nutritional analysis results were compared to the USDA FoodData Central database. This database provides data on the nutrition of common foods consumed in the United States. However, the nutrition data provided by the database only account for cooking, not storage. To the best of the authors’ knowledge, no published information was available regarding the impact of storage on nutritional metrics such as calories and micronutrients for potatoes. Table 6 presents the proximate analysis of the selected variety of russet potato compared to reference literature.

**Table 6:** Proximate composition of Russet potato from various sources.

	Russet Potato	Potatoes, Russet, Flesh and Skin, Raw	Potato
Dry matter %	20.7 ± 0.4	21.4	23.0
Crude Fat %	0.200 ± 0.0	0.370	0.10
Calories cal/gm ADB	3668 ± 0.4	3691	3780
	This study	USDA FoodData	Sharma et al.^58^

### Environmental impact analysis

The environmental impact of household storage and processing was assessed through LCA. This methodology analyzes the effect of a product or process on specific indicators across its entire life span or a specified portion of the life span. In this study, the LCA was conducted following the ISO 14000/44 standards. The impact results were based on OpenLCA v1.11.0 LCA computer modeling system combined with the ReCiPe midpoint and endpoint impact assessment method. The following paragraphs highlight the different stages of LCA; thus, goal and scope, Life Cycle Inventory, Life Cycle Impact Assessment (LCIA) and interpretation of results were performed for this study.

### Goal and scope, functional unit definition

The objective of the LCA was to perform a gate-to-gate environmental impact assessment of household storage and processing of potatoes. The results of the study were intended to provide insights to stimulate consumer practices toward sustainability. The functional unit for the environmental impact assessment is 1 kg of potato processed using different pathways and ready to be eaten by the consumer.

### System boundary and inventory modeling

The system boundary considered is synonymous with the regions of interest proposed in the theoretical framework. In other words, the system boundary captures the consumer purchase from retail points to preparation and consumption. Excluded from the study were the impacts of harvest, preretail transportation, and primary packaging materials. The foreground data were sourced from the laboratory-scale experiment. The LCA of the potato was modeled under three storage conditions for approximately two- and five-week storage times. For the refrigeration condition, the percentage of fridge space dedicated to cooling potatoes was based on the Bureau of Labor Statistics Consumer Expenditure Survey [9]. The Bureau of Labor Statistics Food Expenditure Survey indicates that 6.4% of home food expenditure is on fresh vegetables. The researchers assumed that approximately 50% of the vegetables purchased are stored in the refrigerator, and 5% of fresh vegetable purchases are potatoes. Thus, we estimated that potatoes take up approximately 0.16% of refrigerator space at any time. The low setting on the refrigerator consumes 1.1 kWh/day, while the medium setting consumes 1.5 kWh/day [10].

Similarly, household processing was handled in three pathways: frying, baking, and boiling. First, frying was performed at an average temperature of 140 °C for 7 minutes. Then, the temperature was raised to 208 °C, and samples were fried for an additional 2 minutes, following a recipe from Melchione^48^. Similarly, potatoes were boiled at an average temperature of 100 °C for 15 minutes. Finally, baking was conducted at 120 °C in an oven for 17 minutes, including a 10-minute preheating period. The energy inputs for each cooking method were calculated based on the cooking time and temperature. The equation for the heat transfer is given by:

1 $$Q=m{C}_{p}\Delta T\ldots$$

where $Q$ is the heat transferred in kJ, $m$ is the mass in kg, and ${C}_{p}$ is the specific heat of the material being heated in kJ/kg. K and $\Delta {T}$ is the temperature change in degrees K. The specific heat of the water is 4.18 kJ/kg. K and the specific heat of potatoes to be 3.39 kJ/kg. K, the energy required to raise 1.9 kg of water and 0.93 kg of potatoes from 25 °C (ambient temperature) to 100 °C (boiling point) was estimated to be 1427.8 kJ. Considering the efficiency of the electric burner to be 39%, the total energy required from the burner for the heating stage was 3660.9 kJ (1.01 kWh). After the initial heating stage, the water temperature was maintained for 15 minutes while the heat was lost through convection to the environment. Following the directions posited by^49^, which recommends simmering instead of boiling for 15 minutes, it was assumed that minimal energy losses occur from vaporization and energy is lost exclusively through convection. The convection heat transfer equation is:

2 $$Q={hA}\left({T}_{{{\infty }}}-{T}_{s}\right)\ldots$$

where $h$ is the convection coefficient, $A$ is the surface area of the liquid, ${T}_{\infty }$ is the ambient temperature and ${T}_{s}$ is the surface temperature (average 95°C for simmering water). Additionally, the standard pot size of a four qt saucepan was estimated to be 8 inches (0.2 m) with a surface area of 0.0314 m^2^ for cooking applications. The heat transfer coefficient for ambient air under free air convection was between 5 and 25 W/(m^2^. K); however, an average of 15 W/(m^2^. K) was applied^50^. Therefore, substituting into Eq. 2 yields a heat use of 32.97 W in 15 minutes for a total of 3690 kJ for the entire boiling process.

Similar calculations were repeated for the frying and baking process, which yielded total energy consumption of 8784 kJ (2.4 kWh) and 3888 kJ (1.08 kWh). The baking calculations assumed that the energy consumption was based more on the energy required to maintain the oven temperature than the energy consumed in heating the food.

A conventional mass balance was used to determine the change in mass as applicable to each processing pathway. The biowaste from potato peels was estimated to be 10% of the initial mass after storage. The energy estimations for baking and frying were performed using heat transfer equations based on information from^51–53^. Likewise, the background data were sourced mainly from the Ecoinvent v.3.7.1 database^54^.

### Impact analysis

The impact analysis was conducted using ReCiPe 2016 Midpoint (H), ReCiPe 2016 Endpoint (H), and World 2010 (H/H) normalization methods. The ReCiPe 2016 Midpoint (H) method quantifies the input and output flows for the defined system boundary of each model to 18 impact categories. The impact categories include global warming, carcinogenic potential, acidifications, land use and ozone depletion. Afterward, the midpoint results were translated to endpoint results to reflect three areas of protection: human health, ecosystem quality and resource availability. Furthermore, the endpoint method was utilized to improve the communication of the LCA results and capture the potential damage of each model. At the same time, the midpoint measured the potential impacts^50^. A cutoff of less than 0.1% contribution from the impact category was set for the analysis.

### LCA uncertainty analysis

A sensitivity analysis was performed to provide additional interpretation of the LCA results. Additionally, because LCA involves a significant degree of inherently imprecise estimation, using any LCA software comes with a degree of uncertainty. OpenLCA quantifies this uncertainty in a pedigree matrix that considers contributions to overall uncertainty resulting from five factors: reliability, completeness, temporal correlation, geographic correlation, and other technological correlation. The pedigree matrix returns a geometric standard deviation that can be used to model uncertainty in the system. This uncertainty was modeled using a Monte Carlo simulation feature in OpenLCA, which shows the variation in the impact results from 1000 different simulations in which input variables such as electricity and water use are changed within a 95% confidence interval, and the results are recalculated. The uncertainty simulation also returns a numerical indicator in the coefficient of variation (CV), which gives an indication of the precision of the impact estimate based on the formula.

3 $${CV}=\frac{{std}.{dev}\,\left({A}_{i}\right)}{m\left({A}_{i}\right)}$$

where std. dev (*A*_*i*_) and m (*A*_*i*_) are the standard deviation and mean of the ordered sample, respectively. The CV is a good indicator of the certainty of the results. If the CV is approximately 10% or less, then the impact analysis results for the given inputs are reasonably certain. OpenLCA completes this analysis based on the impact results from the ReCiPe Midpoint (H) analysis.

**Fig. 10:**
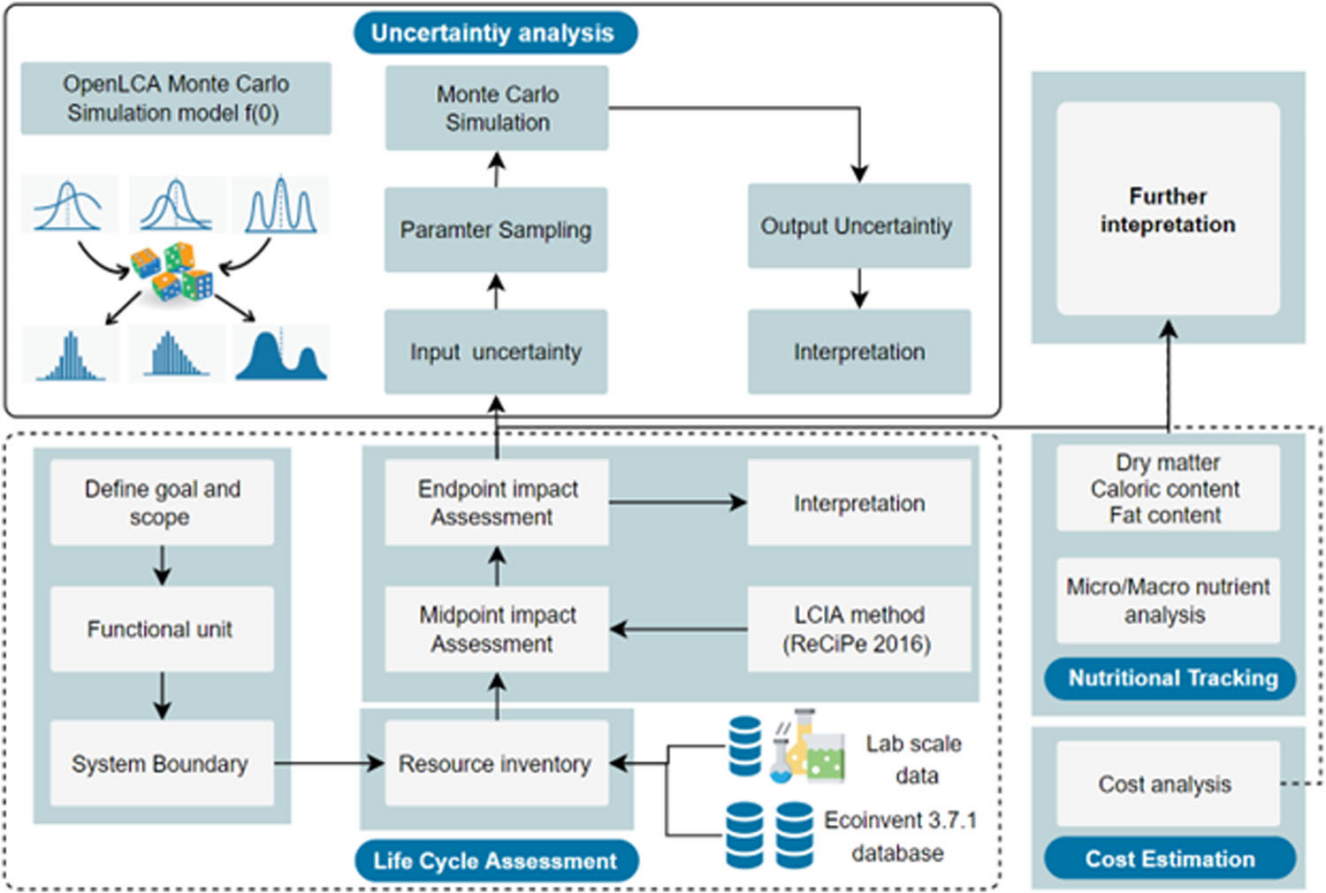
Components of the LCA approach, uncertainty, and sensitivity analysis.

The uncertainty analysis indicates the degree of certainty in the results based on the variability in the possible outputs. On the other hand, sensitivity analysis is conducted to gauge how certain the results are based on the change in response variables corresponding to a change in the input values. In this study, the values for different inputs (specifically cooking temperature and cooking oil use) were changed by ± 10%, and the corresponding difference in the top five normalized impact categories was measured. If the measured difference in the result is 10% or higher, then the impact score is very responsive to the change in input, and the confidence in the certainty of the results is undermined. However, if the difference in the results is low, there is a high degree of certainty. Figure 10 summarizes the compoents of the LCA approach, uncertainty, and sensitivity analysis presented above.

### Decision modeling and trade-off analysis

TOPSIS decision modeling was applied to identify the optimal household storage and processing pathway that provides more minerals and lower environmental impact. TOPSIS ranks the household storage and processing pathways set by selecting the best alternative based on their geometric distance from positive and negative ideal solutions. A detailed description of TOPIS decision modeling is presented extensively by^3,55,56^. To assess the alternative household storage and processing pathways, it was expedient to generate a list of criteria or attributes to guide the decision modeling exercise. Table 7 presents a list of criteria that were developed. From an environmental perspective, human health damage, ecosystem damage and resource depletion are identified. Likewise, from a nutrition perspective, mineral loss/gain for ten macro/micronutrients and the number of calories supplied were selected.

**Table 7:** Criteria for evaluating the performance of household storage and processing pathways.

S/N	Criteria	Description	Objective
	**Environment**		
1	Human health damage	This indicator measures the environmental damage of the modeled product system on the human health	Minimize
2	Resource depletion	This indicator measures USD 2013 loss of resources in a region due to industrial activity	Minimize
3	Ecosystem damage	This indicator measures the level of damage compared to unaffected ecosystems	Minimize
	**Nutrition**		
4	Mineral gain/loss	This indicator measures the mineral loss or gains rate due to the different storage and processing strategies	Maximize/Minimize
5	Calories	This refers to the total amount of energy provided by the processed potato at consumption	Maximized

Additionally, the successful implementation of the TOPSIS model requires criteria weightings. Thus, in this study, environmental sustainability was deemed much more important/better than nutrition or vice versa. Therefore, the verbal preference statement corresponded to a weighting value of [0.8, 0.2] and vice versa. A corresponding comparison weight value of [0.5,0.5] was applied when both criteria were deemed equally important.

### Reporting summary

Further information on research design is available in the Nature Research Reporting Summary linked to this article.

### Supplementary information

Supplementary Document

Reporting Summary

## Data Availability

The authors declare that all data and results generated or analyzed during this study are available from the corresponding author upon request.
