# Supplementary material for: Nutritional-environmental trade-offs in potato storage and processing for a sustainable healthy diet
Source: NPJ Sci Food. 2023 Dec 7;7:63. doi: 10.1038/s41538-023-00237-8 (PMC10703785; doi:10.1038/s41538-023-00237-8)
Supplement: Supplementary file 1 — Supplementary Document [file 41538_2023_237_MOESM1_ESM.docx]

**Toward a Sustainable Healthy Diet: Nutritional-Environmental Trade-offs in Potato Storage and Processing**

*Aubin Payne^1,2^, Ebenezer M. Kwofie^1,2^ , Prince Agyemang^1,2^, and Jamie I. Baum^3,4^*

*^1^Department of Biological and Agricultural Engineering, University of Arkansas, 203 Engineering Hall, Fayetteville, AR 72701, USA.*

*^2^Bioresource Engineering Department, McGill University, Ste-Anne-Bellevue, H9X 3V9, Quebec, Canada*

*^3^Department of Food Science, University of Arkansas, 2650 N. Young Ave.*

*Fayetteville, Arkansas 72704, USA*

*^4^Center for Human Nutrition, University of Arkansas System Division of Agriculture, Fayetteville, AR, 72704, USA*

# Experimental Design

Supplementary Table 1: A factorial design for the lab-scale experiment

| **Pattern** | **Type of storage** | **Processing** | **Time of storage** |
| --- | --- | --- | --- |
| 143 | Ideal | Raw | 33 days |
| 343 | Fridge | Raw | 33 days |
| 232 | Cupboard | Baking | 17 days |
| 133 | Ideal | Baking | 33 days |
| 321 | Unstored | Frying | 0 days |
| 142 | Ideal | Raw | 17 days |
| 313 | Fridge | Boiling | 33 days |
| 332 | Fridge | Baking | 17 days |
| 112 | Ideal | Boiling | 17 days |
| 113 | Ideal | Boiling | 33 days |
| 233 | Cupboard | Baking | 33 days |
| 213 | Cupboard | Boiling | 33 days |
| 341 | Unstored | Raw | 0 days |
| 132 | Ideal | Baking | 17 days |
| 242 | Cupboard | Raw | 17 days |
| 331 | Unstored | Baking | 0 days |
| 333 | Fridge | Baking | 33 days |
| 323 | Fridge | Frying | 33 days |
| 123 | Ideal | Frying | 33 days |
| 322 | Fridge | Frying | 17 days |
| 223 | Cupboard | Frying | 33 days |
| 243 | Cupboard | Raw | 33 days |
| 222 | Cupboard | Frying | 17 days |
| 312 | Fridge | Boiling | 17 days |
| 122 | Ideal | Frying | 17 days |
| 342 | Fridge | Raw | 17 days |
| 311 | Unstored | Boiling | 0 days |
| 212 | Cupboard | Boiling | 17 days |

# Midpoint Environmental Impact Results

Supplementary Table 2: Midpoint results of different processing and storage scenarios (Case of 2 Weeks ±2 days)

| **Indicator** | **BK-US** | **BK-FG** | **BK-ID** | **BK-CP** | **FR-US** | **FR-FG** | **FR-ID** | **FR-CP** | **BL-US** | **BL-FG** | **BL-ID** | **BL-CP** |
| --- | --- | --- | --- | --- | --- | --- | --- | --- | --- | --- | --- | --- |
| FPM, kg PM2.5 eq | 1.75E-03 | 1.79E-03 | 1.91E-03 | 1.75E-03 | 1.51E-02 | 1.51E-02 | 1.52E-02 | 1.51E-02 | 1.67E-03 | 2.81E-02 | 1.83E-03 | 1.67E-03 |
| FRS, kg oil eq | 1.80E-01 | 2.01E-01 | 1.96E-01 | 1.80E-01 | 1.13E+00 | 1.15E+00 | 1.14E+00 | 1.13E+00 | 1.71E-01 | 2.18E+00 | 1.87E-01 | 1.71E-01 |
| FEW, kg 1,4-DCB | 1.76E-02 | 1.81E-02 | 1.86E-02 | 1.76E-02 | 2.97E-01 | 2.98E-01 | 2.98E-01 | 2.97E-01 | 1.71E-02 | 5.33E-01 | 1.81E-02 | 1.71E-02 |
| FWET, kg P eq | 3.85E-04 | 3.96E-04 | 4.16E-04 | 3.85E-04 | 4.64E-02 | 4.65E-02 | 4.65E-02 | 4.64E-02 | 3.68E-04 | 8.19E-02 | 4.00E-04 | 3.68E-04 |
| GLE, kg CO2 eq | 6.11E-01 | 6.82E-01 | 6.66E-01 | 6.11E-01 | 1.51E+01 | 1.51E+01 | 1.51E+01 | 1.51E+01 | 5.84E-01 | 2.71E+01 | 6.37E-01 | 5.84E-01 |
| HCT, kg 1,4-DCB | 2.27E-02 | 2.43E-02 | 2.47E-02 | 2.27E-02 | 5.11E-01 | 5.12E-01 | 5.13E-01 | 5.11E-01 | 2.27E-02 | 9.19E-01 | 2.37E-02 | 2.27E-02 |
| HNCT, kg 1,4-DCB | 3.56E-01 | 3.68E-01 | 3.83E-01 | 3.56E-01 | 2.87E+00 | 2.88E+00 | 2.90E+00 | 2.87E+00 | 3.42E-01 | 5.34E+00 | 3.68E-01 | 3.42E-01 |
| IR, kBq Co-60 eq | 3.21E-03 | 1.68E-02 | 3.40E-03 | 3.21E-03 | 1.99E-01 | 2.12E-01 | 1.99E-01 | 1.99E-01 | 3.36E-03 | 3.75E-01 | 3.41E-03 | 3.36E-03 |
| LUE, m2a crop eq | 4.92E-02 | 5.08E-02 | 4.96E-02 | 4.92E-02 | 1.24E+01 | 1.24E+01 | 1.24E+01 | 1.24E+01 | 4.91E-02 | 2.18E+01 | 4.94E-02 | 4.91E-02 |
| MEE, kg 1,4-DCB | 1.85E-02 | 1.93E-02 | 2.00E-02 | 1.85E-02 | 3.56E-01 | 3.57E-01 | 3.58E-01 | 3.56E-01 | 1.79E-02 | 6.41E-01 | 1.92E-02 | 1.79E-02 |
| ME, kg N eq | 1.36E-04 | 1.37E-04 | 1.38E-04 | 1.36E-04 | 8.78E-03 | 8.78E-03 | 8.79E-03 | 8.78E-03 | 1.34E-04 | 1.55E-02 | 1.37E-04 | 1.34E-04 |
| MAE, kg Cu eq | 4.98E-04 | 5.29E-04 | 5.12E-04 | 4.97E-04 | 2.17E-02 | 2.17E-02 | 2.17E-02 | 2.17E-02 | 5.06E-04 | 3.83E-02 | 5.07E-04 | 5.05E-04 |
| OFHH, kg NOx eq | 1.22E-03 | 1.29E-03 | 1.32E-03 | 1.21E-03 | 1.78E-02 | 1.78E-02 | 1.79E-02 | 1.78E-02 | 1.17E-03 | 3.24E-02 | 1.27E-03 | 1.16E-03 |
| OFTE, kg NOx eq | 1.27E-03 | 1.34E-03 | 1.37E-03 | 1.26E-03 | 1.89E-02 | 1.89E-02 | 1.90E-02 | 1.89E-02 | 1.22E-03 | 3.44E-02 | 1.32E-03 | 1.21E-03 |
| SOEP, kg CFC11 eq | 6.19E-07 | 6.49E-07 | 6.44E-07 | 6.17E-07 | 3.16E-05 | 3.16E-05 | 3.16E-05 | 3.15E-05 | 6.07E-07 | 5.57E-05 | 6.30E-07 | 6.05E-07 |
| TA, kg SO2 eq | 2.10E-03 | 2.21E-03 | 2.28E-03 | 2.10E-03 | 2.05E-02 | 2.06E-02 | 2.06E-02 | 2.05E-02 | 2.01E-03 | 3.79E-02 | 2.19E-03 | 2.01E-03 |
| TSE, kg 1,4-DCB | 7.21E-01 | 7.41E-01 | 7.74E-01 | 7.21E-01 | 1.07E+01 | 1.07E+01 | 1.08E+01 | 1.07E+01 | 6.95E-01 | 1.94E+01 | 7.46E-01 | 6.95E-01 |
| WC, m3 | 2.10E-02 | 2.12E-02 | 2.16E-02 | 2.10E-02 | 3.38E-01 | 3.38E-01 | 3.39E-01 | 3.38E-01 | 2.25E-02 | 6.03E-01 | 2.32E-02 | 2.25E-02 |

Supplementary Table 3: Midpoint results of different storage and processing scenarios (5 weeks ±3 days)

| **Indicator** | **BK-US** | **BK-FG** | **BK-ID** | **BK-CP** | **FR-US** | **FR-FG** | **FR-ID** | **FY-CP** | **BL-US** | **BL-FG** | **BL-ID** | **BL-CP** |
| --- | --- | --- | --- | --- | --- | --- | --- | --- | --- | --- | --- | --- |
| FPM, kg PM2.5 eq | 1.35E-02 | 5.95E-03 | 2.47E-02 | 8.55E-02 | 1.42E-01 | 2.08E-01 | 2.45E-01 | 4.13E-01 | 8.95E-03 | 1.07E-02 | 1.08E-02 | 3.71E-02 |
| FRS, kg oil eq | 1.38E+00 | 3.03E+00 | 2.53E+00 | 8.74E+00 | 1.07E+01 | 1.57E+01 | 1.85E+01 | 3.60E+01 | 9.17E-01 | 1.10E+00 | 1.10E+00 | 3.80E+00 |
| FEW, kg 1,4-DCB | 1.35E-01 | 1.55E-01 | 2.34E-01 | 6.21E-01 | 2.81E+00 | 4.03E+00 | 4.76E+00 | 5.67E+00 | 9.16E-02 | 1.01E-01 | 1.04E-01 | 3.78E-01 |
| FWET, kg P eq | 2.96E-03 | 1.31E-03 | 5.35E-03 | 1.75E-02 | 4.39E-01 | 6.25E-01 | 7.41E-01 | 7.39E-01 | 1.97E-03 | 2.32E-03 | 2.34E-03 | 7.63E-03 |
| GLE, kg CO2 eq | 4.71E+00 | 7.83E+00 | 8.60E+00 | 2.95E+01 | 1.42E+02 | 2.04E+02 | 2.41E+02 | 2.91E+02 | 3.12E+00 | 3.73E+00 | 3.75E+00 | 1.29E+01 |
| HCT, kg 1,4-DCB | 1.75E-01 | 1.39E-01 | 3.18E-01 | 1.06E+00 | 4.83E+00 | 6.93E+00 | 8.19E+00 | 9.97E+00 | 1.22E-01 | 1.38E-01 | 1.39E-01 | 4.80E-01 |
| HNCT, kg 1,4-DCB | 2.74E+00 | 1.65E+00 | 4.89E+00 | 1.52E+01 | 2.71E+01 | 3.95E+01 | 4.65E+01 | 7.49E+01 | 1.83E+00 | 2.12E+00 | 2.15E+00 | 6.96E+00 |
| IR, kBq Co-60 eq | 2.47E-02 | 2.66E-02 | 4.29E-02 | 1.15E-01 | 1.88E+00 | 2.68E+00 | 3.17E+00 | 3.22E+00 | 1.80E-02 | 1.91E-02 | 1.96E-02 | 5.16E-02 |
| LUE, m2a crop eq | 3.79E-01 | 4.43E-01 | 5.98E-01 | 7.25E-01 | 1.17E+02 | 1.67E+02 | 1.97E+02 | 1.88E+02 | 2.63E-01 | 2.56E-01 | 2.71E-01 | 3.28E-01 |
| MEE, kg 1,4-DCB | 1.43E-01 | 1.51E-01 | 2.55E-01 | 7.95E-01 | 3.37E+00 | 4.84E+00 | 5.72E+00 | 7.03E+00 | 9.57E-02 | 1.11E-01 | 1.12E-01 | 4.75E-01 |
| ME, kg N eq | 1.04E-03 | 1.11E-03 | 1.68E-03 | 2.57E-03 | 8.30E-02 | 1.18E-01 | 1.40E-01 | 1.35E-01 | 7.20E-04 | 7.21E-04 | 7.57E-04 | 1.14E-03 |
| MAE, kg Cu eq | 3.83E-03 | 5.89E-03 | 6.29E-03 | 1.18E-02 | 2.05E-01 | 2.92E-01 | 3.45E-01 | 3.44E-01 | 2.71E-03 | 2.72E-03 | 2.84E-03 | 6.33E-03 |
| OFHH, kg NOx eq | 9.39E-03 | 9.61E-03 | 1.69E-02 | 5.57E-02 | 1.68E-01 | 2.42E-01 | 2.86E-01 | 3.87E-01 | 6.26E-03 | 7.34E-03 | 7.42E-03 | 2.43E-02 |
| OFTE, kg NOx eq | 9.78E-03 | 1.01E-02 | 1.76E-02 | 5.69E-02 | 1.78E-01 | 2.57E-01 | 3.04E-01 | 4.05E-01 | 6.53E-03 | 7.61E-03 | 7.70E-03 | 2.48E-02 |
| SOEP, kg CFC11 eq | 4.76E-06 | 6.18E-06 | 8.00E-06 | 1.77E-05 | 2.98E-04 | 4.25E-04 | 5.03E-04 | 5.06E-04 | 3.25E-06 | 3.44E-06 | 3.56E-06 | 7.78E-06 |
| TA, kg SO2 eq | 1.62E-02 | 1.06E-02 | 2.93E-02 | 9.67E-02 | 1.94E-01 | 2.81E-01 | 3.31E-01 | 5.15E-01 | 1.08E-02 | 1.27E-02 | 1.28E-02 | 4.21E-02 |
| TSE, kg 1,4-DCB | 5.55E+00 | 4.27E+00 | 9.87E+00 | 3.01E+01 | 1.01E+02 | 1.46E+02 | 1.72E+02 | 2.23E+02 | 3.72E+00 | 4.27E+00 | 4.34E+00 | 1.34E+01 |
| WC, m3 | 1.62E-01 | 1.59E-01 | 2.66E-01 | 5.11E-01 | 3.20E+00 | 4.57E+00 | 5.40E+00 | 5.86E+00 | 1.21E-01 | 1.24E-01 | 1.30E-01 | 2.36E-01 |

``

# Mineral Variations during storage and processing (Boiling)

Supplementary Table 4 presents the mineral variations after the storage and processing of potato samples from the first batch of experiments conducted. This was conducted between January to March 2022. In this experiment, four bags (5 lb each) of russet potatoes were purchased randomly from a Walmart Supermarket and stored at three storage conditions (Refrigeration-FG, Cupboard-CP, Ideal-ID). Two potato tubers were sampled from the different storage conditions and processed through boiling. From here, two samples from each storage-to-processing combination were collected for duplicate analysis and compared to freshly purchased and processed by boiling (BL-US) and unprocessed potato tuber samples (RW-US).

Supplementary Table 4: Mineral composition for different storage and processing scenarios considering Boiling of potato.

| Minerals (ppm) |  |  | 2 Weeks(±2 days) | | | 5 Weeks (±3 days) | | |
| --- | --- | --- | --- | --- | --- | --- | --- | --- |
|  | RW-US | BL-US | BL-FG | BL-ID | BL-CP | BL-FG | BL-ID | BL-CP |
| Cal | 3668±0.4 | 3711±0.1 | 3732±0.2 | 3714±0.1 | 3697±0.3 | 3782±0.1 | 3682±0.1 | 3581±0.2 |
| Macro minerals | | | | | | | | |
| Ca | 489±6 | 440±2 | 585±1 | 590±4 | 495±6 | 522±5 | 603±6 | 550±5 |
| P | 1803±1 | 1542±0.4 | 2693±1 | 1485±2 | 1741±0.4 | 1745±1 | 1625±1 | 1785±2 |
| K | 12159±7 | 6818±20 | 6233±2 | 5947±0.1 | 5410±3 | 7661±4 | 8060±5 | 6155±3 |
| Na | 270±4 | 138±1 | 170±1 | 121±1 | 123±1 | 166±2 | 156±2 | 117±1 |
| Micro minerals | | | | | | | | |
| Al | 9.5±0.1 | 8.5±0.1 | 8.0±0.1 | 8.4±0.2 | 7.3±0.3 | 8.1±0.2 | 10.1±0.1 | 8.2±0.1 |
| Cu | 2.3±0.1 | 1.5±0.1 | 2.0±0.0 | 1.8±0.0 | 1.8±0.2 | 1.4±0.1 | 2.1±0.2 | 1.9±0.2 |
| Fe | 10.7±0.3 | 7.9±0.0 | 14.7±0.1 | 8.4±0.3 | 19.6±3 | 7.2±1 | 7.8±1 | 8.4±1 |
| Mg | 886±0.1 | 528±3 | 758±2 | 754±2 | 593±2 | 723±3 | 683±4 | 486±2 |
| Mn | 15.2±0.1 | 14.2±0.1 | 22.5±0.2 | 15.3±0.1 | 15.5±0.1 | 14.6±0.2 | 14.4±0.2 | 14.3±0.1 |
| S | 1278±1 | 743±2 | 970±1 | 834±2 | 886±0.5 | 830±1 | 842±2 | 770±2 |

*Cal is calories measured in (Cal/gm ADB). (Two potato tubers were sampled from each storage condition and processed by boiling. After boiling, two samples were collected for each storage-to-boiling combination for duplicate analysis).

# Inter-and Intra-Potato Mineral Variations

Further research was conducted to investigate inter-potato and surface-to-inner flesh mineral variations to augment the current themes reported in the literature. In the second batch of experiments, two bags (5 lb each) of russet potatoes were purchased from Walmart supermarket. Four freshly unstored Russet Potatoes were randomly selected from this batch, and two samples, each from the inner and outer flesh, were collected for duplicate analysis. First, the potato skin was carefully removed with a peeler to a 0.4-0.8 mm depth. Next, the peeled potato tubers were cut longitudinally from top to bottom slices of equal thickness. From here, the surface portions were cut 1.1 to 2 cm from the peeled skin. The second batch of experiments was conducted between March to April 2023.

Supplementary Table 5 below presents the mineral variations within the sampled potatoes. The results in the Table below reveal wide variations for Calcium (426±1 to 251±5 ppm), Phosphorus (6522±20 to 3062±9 ppm), Potassium (39219±50 to 28012±60 ppm), and sodium (408±10 to 105±4 ppm) for the four samples at the surface. Similar observations were made for other microminerals except for Iron. The highest surface mineral concentrations were observed for sample P.Tuber 1, while the lowest was recorded for sample 3. Additionally, it can be observed that a lower mean concentration of macrominerals can be observed in the inner flesh region for potato samples P.Tubers 1, 2, and 4. While the contrary is observed for sample 3, in which higher concentrations of macro minerals are observed at the inner flesh regions. Likewise, in the context of microminerals, Iron concentration is high at the inner flesh region compared to the surface in samples 1 and 2, while the corollary is observed in samples 3 and 4. The highest and least mineral variations were observed for Iron (135% change) and Potassium (3% change) in sample 1.

Supplementary Table 5: Variation in the mineral nutrient concentration of Russet Potatoes **(P.Tuber)**.

|  | **Sample P.Tuber 1** | | | **Sample P.Tuber 2** | | |
| --- | --- | --- | --- | --- | --- | --- |
| **Mineral (ppm)** | **Surface** | **Inner flesh** | **% Change** | **Surface** | **Inner flesh** | **% Change** |
| **Macrominerals** | | | | | | |
| **Ca** | 370±0.0 | 279±4 | 24% | 272±3 | 208±1 | 23% |
| **P** | 6522±20 | 6018±10 | 8% | 3062±9 | 2925±5 | 4% |
| **K** | 39219±50 | 38145±30 | 3% | 33138±30 | 26174±20 | 21% |
| **Na** | 408±10 | 90±1 | 78% | 126±2 | 112±1 | 11% |
| **Microminerals** | | | | | | |
| **Cu** | 2.4±0.1 | 2.1±0.1 | 15% | 1.2±0.1 | 1.0±0.1 | 13% |
| **Fe** | 109±1 | 255±4 | -135% | 60.2±2 | 101.2±1 | -68% |
| **Mg** | 941±9 | 784±4 | 17% | 881±5 | 607±2 | 31% |
| **Mn** | 15.1±2 | 19.0±3 | -26% | 8.3±1 | 3.9±2 | 52% |
|  | **Sample P.Tuber 3** | | | **Sample P.Tuber 4** | | |
|  | **Surface** | **Inner flesh** | **% Change** | **Surface** | **Inner flesh** | **% Change** |
| **Macrominerals** | | | | | | |
| **Ca** | 251±5 | 241±4 | 4% | 426±1 | 264±5 | 38% |
| **P** | 3334±20 | 4498±20 | -35% | 4816±10 | 3525±20 | 27% |
| **K** | 28012±60 | 30954±50 | -11% | 30836±10 | 23066±50 | 25% |
| **Na** | 105±4 | 142±3 | -35% | 122±1 | 96±2 | 21% |
| **Microminerals** | | | | | | |
| **Cu** | 1.0±0.0 | 1.1±0.0 | -19% | 1.2±0.0 | 0.8±0.1 | 32% |
| **Fe** | 86.8±2 | 59.7±2 | 31% | 53.5±2 | 39.7±2 | 26% |
| **Mg** | 601±8 | 682±8 | -13% | 640±1 | 472±7 | 26% |
| **Mn** | 1.0±0.0 | 2.8±0.1 | -188% | 1.0±0.1 | BDL |  |

** All samples were reported on a dry basis. (**Four potato tubers **(P.Tuber)** were randomly selected from the second batch of experiments. For each selected potato tuber, two samples were collected at the inner part and surface flesh for duplicate analysis.)

# Inter and Intra-potato mineral variations during storage

In addition to the above, the authors reproduced the experiment with a similar batch of russet potato samples (Second batch experiment). Russet potatoes from a similar batch (inter potatoes mineral variations analysis in Supplementary Table 5) was stored for two weeks at three different storage conditions**.** Thus, cupboard (16.5 to 20.6℃, 36.4-59.1% RH), refrigeration (3.4 to 12.5℃, 91-100% RH), and Ideal (2.4 to -2.1℃, 60.6-94.3% RH) storage conditions. This time mineral analysis was conducted for the surface and inner part of the potato samples after storage under three different conditions. The results were then compared to the average surface and inner concentration of fresh potato samples in Supplementary Table 5. Supplementary Table 6 presents the mineral distribution at the tuber surface (outer layer), while Supplementary Table 7 presents the variations at the inner layer of the potato. The results are somehow conflicting. It is important to note that the negative percentage change connotes a higher mineral concentration when compared to the reference unstored samples and vice versa. In storage conditions such as refrigeration (3.4 to 12.5$℃$, 91-100% RH), some mineral concentrations are higher than the reference (sample 4), while others are lower than the reference (sample 3). For two potato samples stored during refrigeration, we can observe that the Calcium concentration of sample 3 was reported to be 291±1 ppm. In contrast, sample 4 was reported to be 542±20 ppm, thus a 34% and -23% change, respectively, compared to the average unstored sample. This observation was made for Phosphorus and Iron concentrations during storage in refrigeration. Similarly, for storage under the ideal condition (2.4 to -2.1$℃$, 60.6-94.3% RH), we observe a reported Iron concentration of 50.7±1 ppm for sample 5 and 89.5±1 ppm for sample 6. Thus, a 51% and 13% change in concentration compared to the average unstored samples. Similar observations are made for the potato's inner part (flesh), as presented in Supplementary Table 7.

Additionally, we observe variations between the surface and inner parts of the different samples during storage, ranging from a 76% decrease in concentration (Na concentration in sample 6) to a -83% increase in concentration (P concentration in sample 3). For example, for sample 3 stored during refrigeration, all mineral concentrations at the inner part were reportedly higher than the surface. However, in sample 4, stored during refrigeration, some minerals were higher (Ca and Cu) while others were lower at the surface. The results suggest that the mineral loss and leakages may be attributed to inter-potato and intra-spatial distribution of minerals within potato samples and not necessarily the storage conditions.

Supplementary Table 6: Mineral variations at the surface of potato during storage for two weeks.

|  | **Macro minerals (ppm)** | | | | **Microminerals (ppm)** | | | |
| --- | --- | --- | --- | --- | --- | --- | --- | --- |
|  | **Ca** | **P** | **K** | **Na** | **Cu** | **Fe** | **Mg** | **Mn** |
| **Reference** | 439±5 | 5911±20 | 43735±30 | 254±8 | 1.9±0.0 | 103±3 | 1021±6 | 8.4±2 |
|  | **Cupboard (16.5 to 20.6, 36.4-59.1% RH)** | | | | | | | |
| Sample P.Tuber 1 | 608±9 | 6293±30 | 45816±90 | 336±6 | 1.9±0.6 | 99.9±4 | 1160±10 | 13.2±1 |
| % change | -38% | -6% | -5% | -32% | -1% | 3% | -14% | -56% |
| Sample P.Tuber 2 | -- | -- | -- | -- | -- | -- | -- | -- |
|  | **Refrigeration (3.4 to 12.5, 91-100% RH)** | | | | | | | |
| Sample P.Tuber 3 | 291±1 | 3630±8 | 20497 ±30 | 318±2 | 0.8±0.2 | 41.1±1 | 555±4 | 5.4±0.4 |
| % change | 34% | 39% | 53% | -25% | 56% | 60% | 46% | 36% |
|  |  |  |  |  |  |  |  |  |
| Sample P.Tuber 4 | 542±20 | 7319±6 | 33127±7 | BDL | 1.1±0.1 | 105±3 | 727±6 | 7.1±1 |
| % change | -23% | -24% | 24% | BDL | 41% | -2% | 29% | 15% |
|  | **Ideal (2.4 to -2.1, 60.6-94.3% RH)** | | | | | | | |
| Sample P.Tuber 5 | 521±3 | 4538±9 | 21205±20 | BDL | 0.8±0.2 | 50.7±1 | 671±4 | 8.2±1 |
| % change | -19% | 23% | 52% | BDL | 59% | 51% | 34% | 2% |
|  |  |  |  |  |  |  |  |  |
| Sample P.Tuber 6 | 369±2 | 7956±6 | 35034±10 | 253±3 | 1.2±0.1 | 89.5±1 | 805±2 | 7.2±0.4 |
| % change | 16% | -35% | 20% | 0% | 37% | 13% | 21% | 15% |

(**The negative change in concentration represents an increase in mineral concentration compared to the fresh unstored samples. For each storage condition, two potato tubers were randomly selected. For P.Tuber 2, the analysis results were not interpretable, so this data was not included in the Table. For each selected tuber, two samples were collected at the surface for duplicate analysis).

Supplementary Table 7: Mineral variations in the inner flesh of the potato during storage for two weeks.

|  | **Macro minerals (ppm)** | | | | **Microminerals (ppm)** | | | |
| --- | --- | --- | --- | --- | --- | --- | --- | --- |
|  | Ca | P | K | Na | Cu | Fe | Mg | Mn |
| Reference | 331±2 | 5656±20 | 39447±40 | 147±3 | 1.7±0.1 | 152.0±7 | 849±5 | 8.6±2 |
|  | **Cupboard (16.5 to 20.6, 36.4-59.1% RH)** | | | | | | | |
| Sample P.Tuber 1 | 274±2 | 3270±7 | 21515±20 | BDL | 0.8±0.0 | 49.3±1 | 587±3 | 6.6±1 |
| % change | 17% | 42% | 45% | BDL | 51% | 68% | 31% | 23% |
| Sample P.Tuber 2 | -- | -- | -- | -- | -- | -- | -- | -- |
|  | **Refrigeration (3.4 to 12.5, 91-100% RH)** | | | | | | | |
| Sample P.Tuber 3 | 366±20 | 6647±80 | 32504±20 | BDL | 1.1±0.1 | 62.6±6 | 932±30 | 11.1±3 |
| % change | -11% | -18% | 18% | BDL | 33% | 59% | -10% | -30% |
|  |  |  |  |  |  |  |  |  |
| Sample P.Tuber 4 | 487±6 | 7684±30 | 34949±60 | BDL | 1.1±0.4 | 130.2±2 | 776±9 | 7.2±1 |
| % change | -47% | -36% | 11% | BDL | 38% | 14% | 9% | 17% |
|  | **Ideal (2.4 to -2.1, 60.6-94.3% RH)** | | | | | | | |
| Sample P.Tuber 5 | 314±4 | 4863±10 | 24115±30 | BDL | 0.7±0.3 | 72.0±1 | 611±5 | 8.3±1 |
| % change | 5% | 14% | 39% | BDL | 59% | 53% | 28% | 3% |
|  |  |  |  |  |  |  |  |  |
| Sample P.Tuber 6 | 252±4 | 6724±20 | 30050±50 | 60.8±2 | 0.8±0.3 | 70.6±2 | 675±7 | 7.5±1 |
| % change | 24% | -19% | 24% | 59% | 54% | 54% | 20% | 12% |

**The negative change in concentration represents an increase in mineral concentration compared to the fresh unstored samples. For each storage condition, two potato tubers were selected. For P.Tuber 2, the analysis results were not interpretable, so this data was not included in the Table. For each selected potato tuber, two samples were collected at the inner part for duplicate analysis).
