# Supplementary material for: Nutritional-environmental trade-offs in potato storage and processing for a sustainable healthy diet
Source: NPJ Sci Food. 2023 Dec 7;7:63. doi: 10.1038/s41538-023-00237-8 (PMC10703785; doi:10.1038/s41538-023-00237-8)
Supplement: Supplementary file 2 — Reporting Summary [file 41538_2023_237_MOESM2_ESM.pdf]

nature portfolio

Corresponding author(s): Ebenezer Kwofie (ebenezer.kwofie@mcgill.ca)

Last updated by author(s): Oct 25, 2023

Reporting Summary

Nature Portfolio wishes to improve the reproducibility of the work that we publish. This form provides structure for consistency and transparency in reporting. For further information on Nature Portfolio policies, see our Editorial Policies and the Editorial Policy Checklist.

Statistics

For all statistical analyses, confirm that the following items are present in the figure legend, table legend, main text, or Methods section.

n/a	Confirmed	
☐	☒	The exact sample size (*n*) for each experimental group/condition, given as a discrete number and unit of measurement
☐	☒	A statement on whether measurements were taken from distinct samples or whether the same sample was measured repeatedly
☒	☐	The statistical test(s) used AND whether they are one- or two-sided<br>*Only common tests should be described solely by name; describe more complex techniques in the Methods section.*
☒	☐	A description of all covariates tested
☒	☐	A description of any assumptions or corrections, such as tests of normality and adjustment for multiple comparisons
☐	☒	A full description of the statistical parameters including central tendency (e.g. means) or other basic estimates (e.g. regression coefficient) AND variation (e.g. standard deviation) or associated estimates of uncertainty (e.g. confidence intervals)
☒	☐	For null hypothesis testing, the test statistic (e.g. *F*, *t*, *r*) with confidence intervals, effect sizes, degrees of freedom and *P* value noted<br>*Give P values as exact values whenever suitable.*
☒	☐	For Bayesian analysis, information on the choice of priors and Markov chain Monte Carlo settings
☒	☐	For hierarchical and complex designs, identification of the appropriate level for tests and full reporting of outcomes
☒	☐	Estimates of effect sizes (e.g. Cohen's *d*, Pearson's *r*), indicating how they were calculated

Our web collection on statistics for biologists contains articles on many of the points above.

Software and code

Policy information about availability of computer code

Data collection	No software was used
Data analysis	OpenLCA software Version 1.11.0 for modeling the environmental impact.

For manuscripts utilizing custom algorithms or software that are central to the research but not yet described in published literature, software must be made available to editors and reviewers. We strongly encourage code deposition in a community repository (e.g. GitHub). See the Nature Portfolio guidelines for submitting code & software for further information.

Data

Policy information about availability of data

All manuscripts must include a data availability statement. This statement should provide the following information, where applicable:

- Accession codes, unique identifiers, or web links for publicly available datasets
- A description of any restrictions on data availability
- For clinical datasets or third party data, please ensure that the statement adheres to our policy

The authors declare that all data and results generated or analyzed during this study are available from the corresponding author upon request.

Research involving human participants, their data, or biological material

Policy information about studies with human participants or human data. See also policy information about sex, gender (identity/presentation), and sexual orientation and race, ethnicity and racism.

Reporting on sex and gender	N/A
Reporting on race, ethnicity, or other socially relevant groupings	N/A
Population characteristics	N/A
Recruitment	N/A
Ethics oversight	N/A

Note that full information on the approval of the study protocol must also be provided in the manuscript.

Field-specific reporting

Please select the one below that is the best fit for your research. If you are not sure, read the appropriate sections before making your selection.

☐ Life sciences ☐ Behavioural & social sciences ☒ Ecological, evolutionary & environmental sciences

For a reference copy of the document with all sections, see nature.com/documents/nr-reporting-summary-flat.pdf

Ecological, evolutionary & environmental sciences study design

All studies must disclose on these points even when the disclosure is negative.

Study description	A full factorial design with three storage conditions, three processing pathways and two storage duration as factors. The storage and processing pathways were then modeled in OpenLCA software to assess the environmental impact.
Research sample	In the first experiment, two potato tubers were selected from three storage conditions and processed through three pathways. Then two samples for each storage and processing condition were selected for duplicate analysis. In the second experiment, Four samples (potato tubers) were collected and analyzed at the inner and surface flesh. Additional two potato tubers from each storage condition were sampled and analyzed at the inner and outer flesh area.
Sampling strategy	A full factorial design was created using JMP Software. From the design experiment, different samples from different storage conditions were collected and processed.
Data collection	Storage condition data was collected through a Kestrel DROP S2 Wireless Temperature/Humidity Data Logger. Processing Conditions were recorded using a cooking thermometer.
Timing and spatial scale	In the first experiment, data collection commenced on January 21, 2022 to March 4, 2022. Storage condition data was recorded on in an hourly basis. In the second experiment data collection was done between March and April, 2023.
Data exclusions	Data points for sample 2 (Potato Sample 2 from storage conditions-Cupboard) of the second batch of experiment was excluded from the analysis due the results not being interpretable.
Reproducibility	Attempts to repeat the experiment during the review process were successful
Randomization	Samples for storage were randomly selected to mimic household activities and behavior of consumers.
Blinding	Blinding was not possible since there we no participants or authors who had any conflict in relation to the research

Did the study involve field work? ☐ Yes ☒ No

Reporting for specific materials, systems and methods

We require information from authors about some types of materials, experimental systems and methods used in many studies. Here, indicate whether each material, system or method listed is relevant to your study. If you are not sure if a list item applies to your research, read the appropriate section before selecting a response.

Materials & experimental systems

n/a	Involved in the study
☒	☐ Antibodies
☒	☐ Eukaryotic cell lines
☒	☐ Palaeontology and archaeology
☒	☐ Animals and other organisms
☒	☐ Clinical data
☒	☐ Dual use research of concern
☒	☐ Plants

Methods

n/a	Involved in the study
☒	☐ ChIP-seq
☒	☐ Flow cytometry
☒	☐ MRI-based neuroimaging
